# Microrna profiling analysis of differences between the melanoma of young adults and older adults

**DOI:** 10.1186/1479-5876-8-27

**Published:** 2010-03-19

**Authors:** Drazen M Jukic, Uma NM Rao, Lori Kelly, Jihad S Skaf, Laura M Drogowski, John M Kirkwood, Monica C Panelli

**Affiliations:** 1 Department of Dermatology, University of Pittsburgh School of Medicine, Pittsburgh, Pennsylvania, USA; 2 Department of Pathology, University of Pittsburgh School of Medicine, Pittsburgh, Pennsylvania, USA; 3 Life Technologies, Carlsbad, California, USA; 4 University of Pittsburgh Cancer Institute, Division of Hematology-Oncology Hillman Cancer Center, Pittsburgh, Pennsylvania, USA

## Abstract

**Background:**

This study represents the first attempt to perform a profiling analysis of the intergenerational differences in the microRNAs (miRNAs) of primary cutaneous melanocytic neoplasms in young adult and older age groups. The data emphasize the importance of these master regulators in the transcriptional machinery of melanocytic neoplasms and suggest that differential levels of expressions of these miRs may contribute to differences in phenotypic and pathologic presentation of melanocytic neoplasms at different ages.

**Methods:**

An exploratory miRNA analysis of 666 miRs by low density microRNA arrays was conducted on formalin fixed and paraffin embedded tissues (FFPE) from 10 older adults and 10 young adults including conventional melanoma and melanocytic neoplasms of uncertain biological significance. Age-matched benign melanocytic nevi were used as controls.

**Results:**

Primary melanoma in patients greater than 60 years old was characterized by the increased expression of miRs regulating TLR-MyD88-NF-kappaB pathway (hsa-miR-199a), RAS/RAB22A pathway (hsa-miR-204); growth differentiation and migration (hsa-miR337), epithelial mesenchymal transition (EMT) (let-7b, hsa-miR-10b/10b*), invasion and metastasis (hsa-miR-10b/10b*), hsa-miR-30a/e*, hsa-miR-29c*; cellular matrix components (hsa-miR-29c*); invasion-cytokinesis (hsa-miR-99b*) compared to melanoma of younger patients. MiR-211 was dramatically downregulated compared to nevi controls, decreased with increasing age and was among the miRs linked to metastatic processes. Melanoma in young adult patients had increased expression of hsa-miR-449a and decreased expression of hsa-miR-146b, hsa-miR-214*. MiR-30a* in clinical stages I-II adult and pediatric melanoma could predict classification of melanoma tissue in the two extremes of age groups. Although the number of cases is small, positive lymph node status in the two age groups was characterized by the statistically significant expression of hsa-miR-30a* and hsa-miR-204 (F-test, p-value < 0.001).

**Conclusions:**

Our findings, although preliminary, support the notion that the differential biology of melanoma at the extremes of age is driven, in part, by deregulation of microRNA expression and by fine tuning of miRs that are already known to regulate cell cycle, inflammation, Epithelial-Mesenchymal Transition (EMT)/stroma and more specifically genes known to be altered in melanoma. Our analysis reveals that miR expression differences create unique patterns of frequently affected biological processes that clearly distinguish old age from young age melanomas. This is a novel characterization of the miRnomes of melanocytic neoplasms at two extremes of age and identifies potential diagnostic and clinico-pathologic biomarkers that may serve as novel miR-based targeted modalities in melanoma diagnosis and treatment.

## Background

The incidence of melanoma dramatically increases with age, and accounts for 7% of all malignancies seen in patients between the ages of 15-29 years [1,2]. Despite the fact that almost 450 new patients with melanoma under the age of 20 are diagnosed with melanoma each year in the United States, published reports of this disease in young people have usually been restricted in number and often constitute series from single institutions. Two recently published large studies from the Surveillance Epidemiology and End Results (SEER) and National Cancer Database (NCDB) databases confirmed and expanded previous observations that pediatric/young adult melanoma may be clinically similar to adult melanoma; however some differences in clinical presentation and outcome such as the higher incidence of nodal metastases in children and adolescents with localized disease are evident, particularly in younger patients [1-6].

The outcome of melanoma in the younger, as compared to the older, populations has been shown to differ quite substantially. In the young adult and pediatric population the issue is complicated because of inability even amongst experts to identify conventional melanomas from certain melanocytic neoplasms of uncertain biologic behavior because of subtle overlapping histo-morphological features. Notably in Spitzoid nevi, this subject has been debated since the entity was first described by Sophie Spitz in 1948 [7] because some of these neoplasm have metastasized to regional lymph nodes [8,9]. It has also been recently suggested that the Spitzoid melanocytic neoplasms with nodal metastases may have a better prognosis in young/pediatric age group [10]. In many of the cases, these lesions have been treated as malignant melanomas [11].

**Figure 1:**
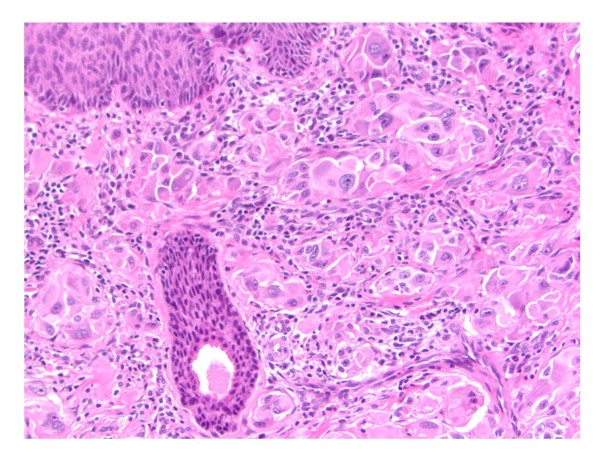
**Atypical Spitz**. Example of atypical Spitz neoplasm of uncertain biological significance.

The aim of this study was to identify the differences between melanoma in young and older adult populations with the ultimate goal of finding useful biomarkers of etiology and outcome at different ages. Therefore we have included some of the Spitzoid melanocytic neoplasms (as a part of the group of patients age less than 30 years old/Mel 30) that have documented sentinel lymph node metastases. (Figure 1).

As Chen summarized [12], the use of DNA microarrays to monitor tumor RNA profiles has defined a molecular taxonomy of cancer, which can be used to identify new drugs and better define prognosis, with the ultimate potential to predict patterns of drug resistance. Cellular behavior is also governed by translational and posttranslational control mechanisms that are not reflected in mRNA profiles of tumor specimens. Since microRNAs regulate gene expression at the post-transcriptional level, the availability of a comprehensive microRNA (miRNAs/miR) expression profile can provide information that is complementary to that derived from mRNA transcriptional profiling. Thus, comprehensive microRNA expression profiling can help to unravel these master regulators of gene expression, which represent a pivotal regulatory network in the transcriptional cell machinery and have been associated with deregulation of immune and cell cycle processes in cancer [13].

MiRNAs are a family of endogenous, small (18-25 nucleotides in length), noncoding, functional RNAs. It is estimated that there may be 1000 miRNA genes in the human genome (Internet address: http://www.sanger.ac.uk/Software/Rfam/mirna/). The latest update of miRBase (Internet address: release 13 March 2009, http://microrna.sanger.ac.uk/sequences/index.shtml) includes more than 1900 annotated miR sequences.

MiRNAs are transcribed by RNA polymerase II or III as longer primary-miRNA molecules, which are subsequently processed in the nucleus by the RNase III endonuclease Drosha and DGCR8 (the "microprocessor complex") to form approximately 70 nucleotide-long intermediate stem-loop structures called "precursor miRNAs" (pre-miRNAs). These pre-miRNAs are transported from the nucleus to the cytoplasm, where they are further processed by the endonuclease Dicer. Dicer produces an imperfect duplex composed of the mature miRNA sequence and a fragment of similar size (miRNA*), which is derived from the opposing arm of the pre-miRNA [14].

Only the mature-miRNA remains stable on the RNA-induced silencing complex (RISC) and induces post-transcriptional silencing of one or more target genes by binding with imperfect complementarity to a target sequence in the 3'-UTR of the target RNA with respect to a set of general rules that are only incompletely determined experimentally and bioinformatically to date [15]. Identification of miRNA targets has been difficult because only the seed sequence, about 6-8 bases of the approximately 22 nucleotides, aligns perfectly with the target mRNA's 3' untranslated region. The remainder of the miRNA may bind perfectly to the target mRNA, but more often it does not [14]. RNA interference and related small RNA mediated pathways are central in the silencing of gene expression, and at least 30% of human genes are thought to be regulated by microRNAs [16]. MiRNAs are expressed in a tissue-specific manner, and can contribute to cancer development and progression. They are differentially expressed in normal tissues and both hematological and solid tumors. In human solid tumors such as hepatocellular carcinoma [17] and ovarian cancer [18], the miRNA expression signature defines neoplasm-specific dys-regulation of specific gene targets.

Despite the hundreds of miRs discovered to date, their biological functions are incompletely understood. Increasing evidence suggests that the expression of miRNAs (miRs) is deregulated in many cancers, and miRs can control cell proliferation, differentiation and apoptosis [19]. The alteration of miR expression may contribute to the initiation and manintanance of tumors as their abnormal levels have important pathogenic consequences: miR overexpression in tumors usually contributes to oncogenesis by downregulating tumor suppressors. For example, the mir-17-miR 92 cluster reduces the transcription factor E2F1 in lymphomas and miR -21 represses the tumor suppressor PTEN in hepatocellular carcinoma. MiRs lost by tumors lead to oncogene overexpression (let -7 loss leads to expression of KRAS, NRAS in lung carcinoma, while miR15a and 16-1 loss leads to expression of BCL-2 in CLL and cyclinD1 in prostate carcinoma [20].

The significance of microRNA differential modulation in the diagnostic and prognostic workup of melanocytic neoplasms, especially in relationship to the age-stratified groups, has not, to our knowledge, been investigated.

In this article, we present profiling results in regard to 666 microRNAs evaluated in melanocytic neoplasms of pediatric and young adults compared with older adults; the results of which emphasize the importance of these master regulators in the transcriptional machinery of melanocytic neoplasms and support the notion that differential levels of expressions of these miRs may contribute to differences in phenotypic and pathologic presentation of melanocytic neoplasms at different ages.

We performed an exploratory analysis of 666 miR on formalin-fixed paraffin-embedded (FFPE)-primary melanoma tissue using the Taqman ^®^TLDA miRNA arrays platform A and B (Applied Biosystems, Foster City, CA, http://www.appliedbiosystems.com) to investigate whether there were differentially expressed miRs between young adult and adult melanoma specimens (including melanocytic neoplasms of uncertain biological potential). The comparative profiling was purposively conducted at extremes of age, <30 and >60 years, to clearly define age groups. Our study represents the first attempt to perform a true intergenerational and comparative microRNA profiling of the primary melanocytic neoplasms of adults and young adults.

We observed distinct miRNA profiles in the primary melanocytic neoplasms of adults and young adults that could also potentially be associated with the clinical parameters of stage and nodal involvement. Our observations represent an important basis for expanded analysis of the etiology and clinico-pathologic spectrum of this disease.

## Materials and methods

### Patient Selection

This study included the utilization of archival melanoma specimens obtained and was approved by the University of Pittsburgh Cancer Institute (UPCI) Internal Review Board (IRB): UPCI reference IRB#: PRO07120294. Archival paraffin blocks of melanocytic neoplasms studied at the UPCI were retrieved from the files of the Health Sciences Tissue Bank (HSTB) database and disbursed by UPCI HSTB according to UPCI-IRB regulations. Ten primary FFPE-tissues (including melanocytic neoplasms of uncertain biological potential) were obtained from two cohorts of patients respectively segregated according to age: Cohort A - > 60 years and Cohort B - <30 years and utilized for microRNA profiling. These two case cohorts were separated by at least 30 years, thereby representing an adequate basis for an intergenerational study.

Additionally, 6 benign nevi were used as homologous controls (3 from adults and 3 from young adult patients, respectively). A total of 26 lesions (20 test specimens + 6 controls) were analyzed. Primary diagnostic workup and verification of the diagnosis of primary neoplasms was performed by two independent reference pathologists.

Total RNA was isolated from all lesions from (at average) 30 5 μm sections obtained specifically from areas that contained at least 70% viable tumor (identified by a pathologist). RNA quality was assessed using Nanodrop (OD 260/280 and 260/230 (Table 1)). The overall microRNA profiling of these two groups (adult and young adult) included a total of 56 Taqman ^® ^microRNA Low density arrays (TLDAs). Each group included 10 melanocytic neoplasm samples (older adult melanoma, AM, pediatric and young adult melanoma PM) and 3 control nevi specimens (adult nevi, AN, pediatric nevi, PN). The assays were run in 3 batches for processing and a calibrator RNA was included in each batch for normalization. For each specimen, 2 TLDA were run, TLDA panel A and TLDA panel B.

**Table 1:** Summary Of RNAs Extracted From FFPE Melanoma And Nevus (Control) Specimens Obtained From Pediatric Or Young Adults < 30 Years Of Age And Older Adults > 60 Years Of Age

Sample ID	Sample Name	FFPE Tissue Type	Percentage Tumor or Nevus	Total RNA yield (ug)	ng/ul RNA	OD 260/280	OD 260/230
TB08-190A	PM7	Mel	80%	2.26	251	1.98	2.02
TB08-192 1H	PM2	Mel	90%	0.45	50.1	1.79	1.47
TB08-239 B	PM3	Mel	80%	0.72	79.61	1.87	1.23
TB09-044B	PM6	Mel	75%	2.03	226	1.94	1.59
TB08-243A	PM8	Mel	85%	1.85	205	1.94	1.95
TB08-231 A	PM4	Mel	75%	0.31	34.97	1.81	1.35
TB08-199D	PM11112	Mel	75%	1.24	103	1.9	1.65
TB08-195 2A	PM5	Mel	80%	0.17	18.69	1.76	1.23
TB08-245D	PM9	Mel	100%	2.37	263	1.94	1.83
TB08-477-478C	PM10	Mel	90%	4.59	255	1.88	1.72
TB08-242A	PN1	Nevus	100%	0.77	85.89	1.86	1.41
TB08-232 2A	PN2	Nevus	100%	2.71	226	1.86	1.56
TB08-188A	PN3	Nevus	100%	0.30	25	1.84	1.45
TB08-236 1L	AM1	Mel	100%	0.93	103.09	1.88	1.6
TB08-180P 1H	AM2	Mel	100%	3.23	269	2	1.86
TB08-217 1D	AM3	Mel	75%	1.42	158.07	1.97	1.64
TB08-223 C	AM10	Mel	70%	0.57	63	1.88	1.72
TB08-181 B	AM4	Mel	95%	11.29	941	1.84	1.35
TB08-211 1J	AM5	Mel	90%	0.66	55	1.89	1.66
TB08-216 F	AM6	Mel	80%	0.46	51.37	1.93	1.59
TB08-219 1G	AM9	Mel	75%	0.47	52	1.89	1.86
TB08-237 1G	AM7	Mel	70%	1.23	136.28	1.85	1.63
TB09-043B	AM8	Mel	90%	2.72	302	1.87	1.17
TB09-003 A	AN1	Nevus	100%	0.90	100	1.99	1.71
TB08-233D	AN2	Nevus	100%	0.36	30	1.93	1.68
TB08-234A	AN3	Nevus	100%	0.12	10.4	1.8	1.22

Top group (PM/PN): young adults <30 yrs old; lower group (AM/AN): adults >60; PM = pediatric and young adult melanoma (<30 yrs); AM = adult melanoma (>60 yrs);PN = pediatric and young adult nevus (<30 yrs); AN = adult nevus (>60 yrs); % tumor refers to the percentage of tumor in the area that was ID & scraped for RNA isolation. Quality of RNA was established by Nanodrop OD reading.

Patient characteristics of specimen groups utilized for class comparison analyses are summarized in Table 2. The pediatric and young adult melanoma (PM) specimens were obtained from 5 males and 5 females, and the 3 control nevi (PN) from 1 male and 2 females. Patient PM8 had a Spitzoid neoplasm of uncertain malignant potential, PM5 was classified as stage 0, 6 PM patients were classified as Stage I or II (PMs 11112, 3, 4, 6, 7_(Tstage)_, 10), PM2 was classified as Stage III and PM9 as Stage IV.

**Table 2:** Patients Characteristics

Sample name	Mel 60/30 or Nevus 60/30	Age	Age range	Gender	Diagnosis	Site	T Stage	N Stage	M Stage	**Stage Group at Diagnosis- AJCC 6th Ed**.
PM7	Mel 30	21	20-29	M	Melanoma, invasive and insitu, arising in association with a nevus	Trunk	cT1*	pN0	cM0	Unknown

PM2	Mel 30	26	20-29	M	Superficial spreading melanoma, invasive and in situ	Back	pT1b	pN1a	cM0	3B

PM3	Mel 30	26	20-29	F	Melanoma, superficial spreading in radial growth phase & vertical, epithelioid, nevoid and balloon cell	Scapula	pT2b	pN0	cM0	2A

PM6	Mel 30	28	20-29	F	Superficial spreading melanoma, invasive	Thigh	pT1b	pN0	cM0	1B

PM8	Mel 30	28	20-29	M	Highly atypical spitzoid neoplasm	Arm	n/a	n/a	n/a	n/a

PM4	Mel 30	28	20-29	F	Superficial spreading melanoma, invasive	Shin	pT1a	pN0	cM0	1A

PM11112	Mel 30	29	20-29	F	Superficial spreading (Spitzoid) melanoma, insitu & invasive	Thigh	pT1a	pN0	cM0	1A

PM5	Mel 30	29	20-29	M	Melanoma in situ (arising in compound melanocytic nevus)	Abdomen	pTis	cN0	cM0	0

PM9	Mel 30	29	20-29	F	Invasive and in situ melanoma, nodular. Note: Description of superficial spreading also in synopsis but registry only codes final diagnoses.	Buttock	pT4b	pN3	cM1c	4

PM10	Mel 30	29	20-29	M	Superficial spreading melanoma, insitu and invasive	Scalp	pT1a	cN0	cM0	1A

PN1	Nevus 30	12	10-19	F	Compound, predominantly intradermal melanocytic nevus	Forehead	n/a	n/a	n/a	n/a

PN2	Nevus 30	14	10-19	M	Compound predominantly intradermal melanocytic nevus with architectural features of congenital onset	Scalp	n/a	n/a	n/a	n/a

PN3	Nevus 30	26	20-29	F	Compound melanocytic nevus with features of a congenital nevus, architectural disorder and mild cytologic atypia (aka Clark's nevus with features of congenital onset).	Back	n/a	n/a	n/a	Unknown

AM1	Mel 60	64	60-69	F	Melanoma, invasive, nevoid type.	Leg	pT2a	pN0	cM0	1B

AM2	Mel 60	69	60-69	M	Superficial spreading (outside path) and Nevoid Melanoma, invasive	Ear	pT4b	pN3	cM0	3C

AM3	Mel 60	69	60-69	M	Desmoplastic melanoma, invasive	Forehead	pT3a	pN0	cM0	2A

AM10	Mel 60	72	70-79	M	Malignant melanoma in situ arising in a compound dysplastic nevus	Back	pTis	cN0	cM0	0

AM4	Mel 60	73	70-79	M	Nodular melanoma, invasive and insitu	Calf	pT4b	pN3	cM0	3C

AM5	Mel 60	78	70-79	F	Melanoma, insitu and invasive	Foot	pT2b	pN2c	cM0	3B

AM6	Mel 60	79	70-79	M	Lentingo malignant melanoma in situ with focus invasive melanoma	Back	pT1a	cN0	cM0	1A

AM9	Mel 60	79	70-79	M	Invasive melanoma (&Melanoma in Situ arising in a background of dysplastic nevus	Back	pT1a	cN0	cM0	1A

AM7	Mel 60	82	80-89	F	Desmoplastic melanoma with associated lentiginous component	Arm	pT4a	pN0	cM0	2B

AM8	Mel 60	86	80-89	M	Nodular melanoma (3% in situ)	Flank	pT2a	cN0	cM0	1B

AN1	Nevus 60	62	60-69	F	Compound, predominantly intradermal melanocytic nevus with architectural features of congenital onset	Back	n/a	n/a	n/a	n/a

AN2	Nevus 60	63	60-69	M	Compound predominantly intradermal melanocytic nevus with architectural features of congenital onset	Flank	n/a	n/a	n/a	n/a

AN3	Nevus 60	68	60-69	M	Compound melanocytic nevus with moderate cytological atypia and congenital features.	Deltoid	n/a	n/a	n/a	n/a

PM = pediatric and young adult melanoma (<30 yrs);AM = adult melanoma (>60 yrs);PN = pediatric and young adult nevus(<30 yrs); AN = adult nevus(>60 yrs); Mel 60: adult melanoma (>60 yrs); Mel 30: pediatric and young adult melanoma (<30 yrs); Nevus 60: adult nevus(>60 yrs); Nevus 30: pediatric and young adult nevus(<30 yrs). TNM Staging:regardless of year of diagnosis, all cases staged according to AJCC 6th Edition. P:pathologic staging; c: clinical staging. * Not able to stage T further as Clarks level missing in original path report.

The adult melanomas (AM) were obtained from 3 female patients and 7 male patients, the nevi (AN) were obtained from 1 female and 2 male patients. AM10 was classified as stage 0 (AM10), 6 AM patients as Stage I or II (AM1, 3, 6, 7, 8, 9) and 3 AM patients as Stage III (AM2, 4, 5).

Two patients PM patients (PM2 and PM9) and 3 patients AM patients (AM2, AM4, AM5) had melanoma which spread to the lymph nodes.

### Taqman^® ^microRNA Low density arrays (TLDA)

**Figure 2:**
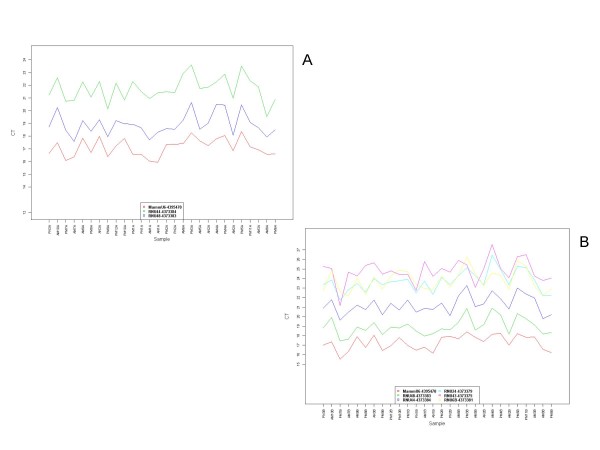
**Engogenous Control Profiles**. **A: **endogenous controls of TLDA panel A profiled across all specimens. **B: **endogenous controls of TLDA panel B profiled across all specimens. The Mammalian U6 assay was selected for data normalization. Endogenous controls in panel A included MammU6-4395470, RNU44-4373384, RNU48-4373383. Endogenous control in panel B included MammU6-4395470, RNU44-4373384, RNU48-4373383, RNU244373379, RNU434373375, RNU6B-4373381

The ABI Taqman^® ^microRNA Low density arrays (TLDA, Applied Biosystems, Foster City, CA, http://www.appliedbiosystems.com) were selected as the platform for microRNA melanoma profiling (additional file 1). This platform consists of 2 arrays: TLDA panel A (377 functionally defined microRNAs) and TLDA panel B (289 microRNAs whose function is not yet completely defined) for a total of 666 microRNA assays. Each array/panel includes, among other endogenous controls, the mammalian U6 (MammU6) assay that is repeated four times on each card as a positive control as well as an assay unrelated to mammalian species, ath-miR159a, as negative control (Figure 2). This platform represented the most comprehensive Taqman Low Density Array (TLDA) for global screening of miRs for which commercially available primer-probe sets existed that were extensively validated.

### Isolation of RNA, Reverse Transcription, Preamplification and Taqman PCR

Total RNA was isolated from FFPE-tissue utilizing a modified RecoverALL (Recover All Ambion #AM1975) protocol for isolation of RNA from paraffin slide sections. In brief, using a scalpel blade (#15) wetted in xylene, areas containing >70% tumor were excised from thirty 5 um paraffin tissue sections and placed in an microcentrifuge tube containing 1 ml of xylene, vortexed and incubated at 50°C for 3 minutes to melt the paraffin. The material was then centrifuged at 14,000 rpm for 5-10 min at room temperature. The xylene was then removed using a 1 ml pipette and the pellet was washed 3 times with 1 ml of 100% room temperature-ethanol. The pellet was then air-dried at room temperature for 15 minutes. Following deparaffinization, tissue was protease digested by incubating the pellet in 400 ul digestion buffer and 4 ul protease at 50°C for 3 hours. For RNA isolation, 480 ul of isolation additive was added to the sample, followed by vortexing and addition of 1.1 ml of 100% ethanol. The mixture was then loaded onto a prepared filter and collection tube according to the manufacturer-supplied procedure. Flow through was discarded and filter washed with wash buffer. Nuclease digestion and final RNA purification was carried over as follows. Sixty ul DNase master mix (containing 6 ul 10× DNase buffer, 4 ul DNase, 50 ul nuclease free water) was added to the center of the filter and incubated for 30 minutes at room temperature. The filter was subsequently washed according to the manufacturer's protocol, and RNA was eluted twice with 30 ul preheated nuclease-free water. RNA quality and quantity was measured by Nanodrop technology.

RNA was further purified and concentrated by precipitation for 1 hour at -70°C using 1/10 volume ammonium acetate, 1 ul glycogen (5 ug/ul) and 2.5 volume 100% ethanol. RNA was then washed, dried and resuspended in 12-15 ul nuclease-free water.

RNA reverse transcription was accomplished according to the ABI microRNA TLDA Reverse Transcription Reaction protocol. In brief, the Megaplex RT Primers, TaqMan^® ^MicroRNA Reverse Transcription Kit components and MgCl_2 _were thawed on ice. Two master mixes per specimen, one for each TLDA panel (panel A and panel B) consisting of 0.80 ul MegaPlex RT primers (10×), 0.20 ul dNTPs with dTTP (100 mM), 1.50 ul MultiScribe™ ReverseTranscriptase (50 U/μL), 0.80 ul 10? RT Buffer, 0.90 ul MgCl_2 _(25 mM), 0.10 ul RNase Inhibitor, 0.20 ul nuclease-free water (20 U/μL) were prepared. Three μL (30 ng) total RNA (or 3 uL of water for the No Template Control reactions) were loaded into appropriate wells of a 96-well plate containing 4.5 uL RT reaction mix and incubated on ice for 5 min. The following thermal cycling conditions were used in the ABI 9700 thermal cycler: standard or max ramp speed, 16°C 2 min, 42°C 1 min 40 cycles, 50°C 1 sec, hold 85°C 5 min, hold 4°C.

The cDNA product (2.5 ul per specimen) was preamplified according to the ABI TLDA preamplification protocol. A total of 22.5 ul of pre-amplification reaction mix consisting of 12.5 ul TaqMan^® ^PreAmp Master Mix (2×); 2.5 ul Megaplex™ PreAmp Primers (10×); 7.5 ul nuclease-free water was prepared and added to the cDNA product in a 96-well optical plate sealed with MicroAmp^® ^Clear Adhesive Film (ABI PN #4306311). The plate was spun briefly and incubated on ice for 5 min. The preamplifcation was conducted in the ABI 9700 thermal cycler using standard ramp speed and the following thermal cycling conditions: hold 95°C10 min; hold 55°C 2 min; hold 72°C 2 min; 12 cycle at 95°C 15 sec and 60°C 4 min; hold 4°C forever.

The preamplified product was diluted with 75 uL of 0.1× TE pH 8.0 mixed, briefly centrifuged and stored at -25°C before TaqMan Real Time assay.

TLDA TaqMan Real Time Assay was set up for each sample as follows: 450 μl of TaqMan^® ^Universal PCR Master Mix-No AmpErase^® ^UNG (2×) were added to 9 μl of diluted PreAmp product in a 1.5-mL microcentrifuge tube containing 441 ul of nuclease-free water. The reaction was mixed six times by inverting the tube and then briefly centrifuged.

One hundred ul of the PCR reaction mix were loaded into each port of the TLDA array.

The TLDA plate was centrifuged with 9 up and down ramp rates at 1200 rpm for 1 min and loaded into the 7900 HT Sequence Detection System using the 384-well TaqMan Low Density Array default thermal-cycling conditions.

### Data Analysis

TLDA were run in the 7900 HT Sequence Detection system. The ABI TaqMan SDS v2.3 software was utilized to obtain raw C_T _values. To review results, the raw C_T _data (SDS file format) were exported from the Plate Centric View into the ABI TaqMan RQ manager software. Automatic baseline and manual CT were set to 0.2 for all samples.

The data discussed in this publication have been deposited in NCBI's Gene Expression Omnibus (GEO) and are accessible through GEO Series accession number **GSE19229 **(Internet address: http://www.ncbi.nlm.nih.gov/geo/query/acc.cgi?acc=GSE19229).

#### Statistical analysis of TLDA

The global data set of 666 miRs was used for analysis.

Data analysis used two different methods. The first method (Analysis I) utilized ABqPCR package (kindly provided and supported by Dr. Jihad S. Skaf, SOLiD Next Generation Sequencing Specialist Applied Biosystems. This software utilizes values obtained from relative quantification of miRs for class comparisons and generation of fold changes (FC values).

The cutoff P value for the Student T test performed in ABqPCR was set at < 0.05 level of significance. MammU6 was used as an endogenous control (Figure 2). Fold changes (FC values) were calculated from the raw Cycle Threshold (C_T_) values by the DataShop software according to the following formula:

FC = 2 - (delta delta C_T_)

[delta][delta] C_T _= [delta] C_T_, sample - [delta] C_T_, reference

delta delta C_T _= [C_T _Mel - C_T _MammU6] - [C_T _Nevus - C_T _MammU6]

In which" [delta] C_T_, sample" is the C_T _value for any specimen normalized to the endogenous housekeeping MammU6, and " [delta] C_T_, reference" is the C_T _value for the calibrator (TB-08-242A, PN1), also normalized to the endogenous housekeeping miR. PN1 was chosen as calibrator for all samples.

The second method (Analysis II) utilized BRB Tools [21]. Input data for class comparison, permutations and prediction analysis consisted of the miR expression C_T _values normalized to the endogenous housekeeping MammU6 (C_T_, sample - C_T_, MammU6).

### Class comparison univariate and multivariate analysis

Class comparison between the various groups (Mel 60, Mel 30, Nevus 60, Nevus 30) was performed along with univariate Two-sample T-test. The nominal significance level of each univariate test was 0.05. The global data set of 666 miRs was used for analysis. MiRs were considered statistically significant if their p-value was ≤ 0.05. A stringent significance threshold was used to limit the number of false positive findings.

We also performed a global test of whether the expression profiles differed between the classes by permuting the labels of which arrays corresponded to which classes. For each permutation, the p-values were re-computed and the number of genes significant at the 0.001 level was noted. The significance level of the global test was the proportion of the permutations that gave at least as many significant miRs as were given with the actual data.

We identified miRs that were differentially expressed among the two classes using a multivariate permutation test [22,23]. We used the multivariate permutation test to provide 90% confidence that the false discovery rate was less than 10%. The false discovery rate is the proportion of the list of miRs claimed to be differentially expressed that are false positives. The test statistics used are random variance t-statistics for each miR [24]. Although t-statistics were used, the multivariate permutation test is non-parametric and does not require the assumption of Gaussian distributions.

### Multidimensional scaling/PCA analysis

BRB-ArrayTools was used to perform multi-dimensional scaling analysis (MDA) of the miRs expressed in melanoma and nevi samples. In a 3-dimensional representation, the samples with very similar expression profiles are displayed close together. The MDA was computed using Euclidean distance, hence it was equivalent to a principal component analysis (PCA). BRB-ArrayTools utilized the first three principal components as the axes for the multi-dimensional scaling representation. The principal components are orthogonal linear combinations of the miRs. That is, they represent independent perpendicular dimensions that are rotations of the miR axes. The first principal component is the linear combination of the miRs with the largest variance over the samples of all such linear combinations. The second principal component is the linear combination of the miRs that is orthogonal (perpendicular) to the first and has the largest variance over the samples of all such orthogonal linear combinations, and so on. The samples were first centered by their means and standardized by their norms, and then the multi-dimensional scaling components were computed using a Euclidean distance on the resulting centered and scaled sample data. The statistical significance test was based on a null hypothesis that the expression profiles came from the same multivariate Gaussian (normal) distribution. A multivariate Gaussian distribution is a unimodal distribution that represents a single cluster.

### Class Prediction

We developed models for utilizing the miR expression profiles to predict the class of future samples. We developed models based on the Compound Covariate Predictor [25], Diagonal Linear Discriminant Analysis, Nearest Neighbor Classification [26], and Support Vector Machines with linear kernel [27]. The models incorporated genes that were differentially expressed among genes at the 0.001 significance level, as assessed by the random variance t-test [24]. We estimated the prediction error of each model using leave-one-out cross-validation (LOOCV) as described by Simon et al. [28].

For each LOOCV training set, the entire model-building process was repeated, including the gene selection process. We also evaluated whether the cross-validated error rate estimate for a model was significantly less than one would expect from random prediction. The class labels were randomly permuted and the entire LOOCV process was repeated. The significance level is the proportion of the random permutations that gave a cross-validated error rate no greater than the cross-validated error rate obtained with the real data. A total of 1000 random permutations were used.

#### Hierarchical clustering analysis

The log (base 2) transformed FC expression values or the MammU6 normalized C_T _values were used to visualize modulation of miRs in heat maps by hierarchical clustering analysis according to Eisen [29].

*Mining analysis *was conducted utilizing the following open access microRNA data bases with the following internet addresses:

**Mirdata base **[30]: http://microrna.sanger.ac.uk/sequences/

**MicroCosm Targets Version 5 **http://www.ebi.ac.uk/enright-srv/microcosm/htdocs/targets/v5/

**Entrez cross data base search**: http://www.ncbi.nlm.nih.gov/sites/gquery;

**Entrez Gene: **http://www.ncbi.nlm.nih.gov/sites/gquery

**Gene Cards: **http://www.genecards.org/

**Pic Tar **data base: http://pictar.mdc-berlin.de/cgi-bin/PicTar_vertebrate.cgi was used to for identification of predicted miR target

**Mir2Disease database **[31]: is a manually curated database for microRNA deregulation in human disease and was used to identify the deregulation of specific miRs across different diseases http://www.mir2disease.org/

**The Melanoma Molecular Map project **http://www.mmmp.org/MMMP/ is a multiinteractive data base for research on melanoma biology and treatment. It was used to mine the miRNAs reported to date to be differentially modulated in melanoma compared to normal tissue.

## Results

Primary melanoma lesions, separated according to two age groups (< 30 and > 60 years old), were utilized for microRNA profiling. Each group included 10 samples of melanoma (older adult melanoma, AMs, and pediatric to young adult melanoma, PMs) and 3 each control nevi specimens (adult nevi, ANs, and pediatric-young adult nevi, PNs, respectively). For each specimen 2 TLDA were run, TLDA panel A and TLDA panel B. Patient characteristics are displayed in Table 2, which defines the groups of specimens utilized for the class comparison analyses.

**Figure 3:**
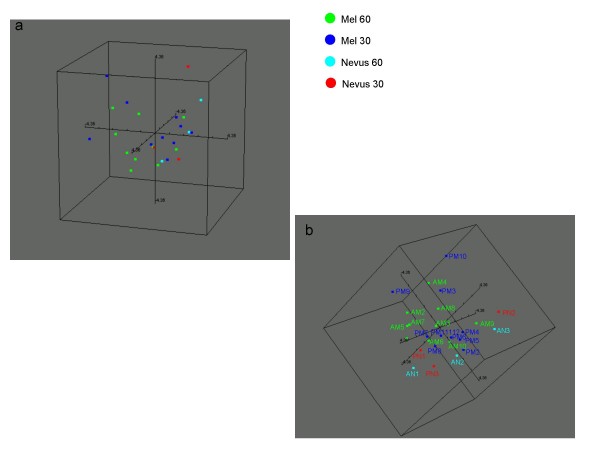
**Multidimensional scaling analysis based on 666 miRs across all samples**. **a) **Multidimensional scaling analysis (MSA) based on the 666 miRs across all samples by analysis II (BRB tools/MDS **b) **MSA represented in a) rotated in space to enhance the visualization of melanomas and nevi controls.

Multidimensional Scaling Analysis was performed on the global miR data set utilized in analysis II of 666 miRs across all samples to visualize similarities and dissimilarities between AMs, PMs and respective control nevi. (Figure 3a and 3b). The majority of PMs clustered in space in close proximity to the nevi controls (PNs and also ANs) (Figure 3b). Interestingly three adult melanomas (AM 6, 9, 10) grouped closely to the young adult cases and nevi; AM9 and AM10 both developed from dysplastic nevi. Furthermore, 3 young adult cases (PM 3, 9, 10) grouped with the adult cases. All three cases were characterized by superficial spreading. PM9, the case with the highest stage (Stage IV), grouped further away not only from the other young adult but also from the adult cases.

Class comparison analyses were conducted between the two major groups of 10 primary melanomas each and the respective nevi controls: 10 AM, 3 AN, 10 PM and 3 PN. Utilizing the first of the two approaches described in the analysis section (relative quantification method), 35 miRs were found to be differentially expressed between AMs and PMs (Mel 60 vs Mel 30), (Table 3); 36 miRs were significantly differentially expressed between ANs and AMs (Nevus 60 vs Mel 60, Table 4); 39 miRs between PNs and PMs (Nevus 30 vs Mel 30, Table 5); 2 differentially expressed between ANs vs PNs (Nevus 60 vs Nevus 30, Table 6) at the p < 0.05 level of significance. Results from the relative quantification approach were compared with those obtained from normalized-absolute quantification values of miR expression. Twenty miRs were identified by both methods to be differentially expressed between Nevus 60 vs Mel 60, 17 between Nevus 60 vs. Mel 60, 10 between Nevus 30 vs Mel 30 and 1 between Nevus 60 vs Nevus 30 (Table 7).

**Table 3:** Mirs Significantly Differentially Expressed Between Older Adult Melanoma (Mel 60) And Pediatric And Young Adult Melanoma (Mel 30)

Array A Hsa-miR Name-Assay#	FC (MEL60/MEL30)	Log2(FC)	p value	FDR (BH)	FC Bin
**hsa-miR-204-4373094**	34.6805	5.1161	0.0007	0.1571	FC > 4
**hsa-miR-199a-5p-4373272**	4.3354	2.1162	0.0024	0.2701	FC > 4
**hsa-miR-211-4373088**	0.2785	-1.8441	0.0044	0.2701	FC 2.0-4.0
**hsa-miR-574-3p-4395460**	1.8143	0.8594	0.0053	0.2701	FC 1.6-2.0
**hsa-miR-449a-4373207**	0.3750	-1.4150	0.0057	0.2701	FC 2.0-4.0
**hsa-miR-455-5p-4378098**	0.4594	-1.1221	0.0070	0.2788	FC 2.0-4.0
**hsa-miR-337-5p-4395267**	2.6855	1.4252	0.0167	0.4867	FC 2.0-4.0
**hsa-let-7b-4395446**	1.9118	0.9349	0.0212	0.4867	FC 1.6-2.0
hsa-miR-140-3p-4395345	1.6343	0.7087	0.0221	0.4867	FC 1.6-2.0
hsa-miR-330-3p-4373047	1.9706	0.9786	0.0229	0.4867	FC 1.6-2.0
**hsa-miR-489-4395469**	1.8103	0.8563	0.0251	0.4867	FC 1.6-2.0
**hsa-miR-24-4373072**	0.6601	-0.5992	0.0264	0.4867	FC 1.2-1.6
**hsa-miR-146b-3p-4395472**	2.6336	1.3970	0.0283	0.4867	FC 2.0-4.0
hsa-miR-125b-4373148	1.8045	0.8516	0.0292	0.4867	FC 1.6-2.0
hsa-miR-192-4373108	0.6908	-0.5336	0.0334	0.4867	FC 1.2-1.6
**hsa-miR-10b-4395329**	2.2070	1.1421	0.0341	0.4867	FC 2.0-4.0
hsa-miR-199b-5p-4373100	2.3762	1.2486	0.0348	0.4867	FC 2.0-4.0
hsa-miR-19b-4373098	0.5745	-0.7996	0.0369	0.4873	FC 1.6-2.0
hsa-miR-423-5p-4395451	2.0952	1.0671	0.0398	0.4909	FC 2.0-4.0
hsa-miR-20a-4373286	0.5834	-0.7775	0.0421	0.4909	FC 1.6-2.0
hsa-miR-9-4373285	3.4546	1.7885	0.0433	0.4909	FC 2.0-4.0
					
**Array B Hsa-miR Name-Assay#**	**FC (MEL60/MEL30)**	**Log2(FC)**	**p value**	**FDR (BH)**	**FC Bin**

**hsa-miR-30aSTAR-4373062**	2.2183	1.1494	0.0000	0.0021	FC 2.0-4.0
**hsa-miR-10bSTAR-4395426**	1.7444	0.8027	0.0022	0.0739	FC 1.6-2.0
**hsa-miR-30eSTAR-4373057**	1.6826	0.7507	0.0026	0.0739	FC 1.6-2.0
hsa-miR-409-3p-4395443	2.1484	1.1032	0.0049	0.1038	FC 2.0-4.0
**hsa-miR-29cSTAR-4381131**	2.2418	1.1647	0.0069	0.1151	FC 2.0-4.0
**hsa-miR-125b-1STAR-4395489**	2.7217	1.4445	0.0096	0.1341	FC 2.0-4.0
hsa-miR-432-4373280	2.6512	1.4066	0.0157	0.1808	FC 2.0-4.0
**hsa-miR-505STAR-4395198**	2.2251	1.1539	0.0193	0.1808	FC 2.0-4.0
**hsa-miR-944-4395300**	0.4042	-1.3068	0.0204	0.1808	FC 2.0-4.0
hsa-miR-766-4395177	2.6347	1.3976	0.0215	0.1808	FC 2.0-4.0
**hsa-miR-214STAR-4395404**	1.7814	0.8330	0.0252	0.1926	FC 1.6-2.0
**hsa-miR-99bSTAR-4395307**	1.4101	0.4958	0.0285	0.1993	FC 1.2-1.6
hsa-miR-572-4381017	0.4892	-1.0314	0.0411	0.2653	FC 2.0-4.0
hsa-miR-768-3p-4395188	1.2722	0.3474	0.0483	0.2896	FC 1.2-1.6

Array A: TLDA panel A (377 functionally defined microRNAs) array B: TLDA panel B (290 MicroRNAs whose function is not yet completely defined) TLDA A and B totaled 667 microRNA assays. FC: fold change; Pvalue student T test ≤ 0.05; FDR: false discovery rate; FC bin: Range of fold change. MirRs in **bold **font were found to be significantly differentially expressed between the two groups by the relative quantification (ABqPCR software-Analysis I) based method and by Class Comparison (BRB tools-Analysis II) based on absolute CT values normalized to endogenous control MammU6 (see materials and methods). N/A: not applicable.

**Table 4:** Mirs Significantly Differentially Expressed Between Adult Nevus (Nevus 60) And Adult Melanoma (Mel 60)

Array A Hsa-miR Name-Assay#	FC (NEVUS60/MEL60)	Log2(FC)	p value	FDR (BH)	FC Bin
**hsa-miR-211-4373088**	23.2024	4.5362	0.0000	0.0009	FC > 4
**hsa-miR-455-5p-4378098**	4.0390	2.0140	0.0001	0.0099	FC > 4
**hsa-miR-891a-4395302**	11.9232	3.5757	0.0010	0.0768	FC > 4
**hsa-miR-532-3p-4395466**	2.0532	1.0379	0.0017	0.0997	FC 2.0-4.0
**hsa-miR-888-4395323**	9.6379	3.2687	0.0023	0.1103	FC > 4
**hsa-miR-574-3p-4395460**	1.7254	0.7869	0.0037	0.1287	FC 1.6-2.0
**hsa-miR-510-4395352**	11.7097	3.5496	0.0038	0.1287	FC > 4
hsa-miR-382-4373019	0.0794	-3.6541	0.0049	0.1454	FC > 4
**hsa-miR-98-4373009**	0.0532	-4.2327	0.0099	0.2571	FC > 4
**hsa-miR-576-3p-4395462**	0.2275	-2.1362	0.0109	0.2571	FC > 4
**hsa-miR-539-4378103**	0.2609	-1.9384	0.0118	0.2571	FC 2.0-4.0
hsa-miR-509-5p-4395346	5.3581	2.4217	0.0173	0.3251	FC > 4
hsa-miR-424-4373201	0.2554	-1.9691	0.0177	0.3251	FC 2.0-4.0
hsa-miR-513-5p-4395201	3.7696	1.9144	0.0208	0.3553	FC 2.0-4.0
hsa-miR-493-4395475	0.1723	-2.5369	0.0270	0.4147	FC > 4
**hsa-miR-197-4373102**	2.5425	1.3462	0.0290	0.4147	FC 2.0-4.0
hsa-miR-508-3p-4373233	3.3230	1.7325	0.0295	0.4147	FC 2.0-4.0
**hsa-miR-146b-5p-4373178**	0.3392	-1.5599	0.0382	0.5068	FC 2.0-4.0
**hsa-miR-23b-4373073**	3.4283	1.7775	0.0414	0.5208	FC 2.0-4.0
hsa-miR-362-5p-4378092	0.5702	-0.8104	0.0442	0.5208	FC 1.6-2.0
**hsa-miR-223-4395406**	0.3426	-1.5453	0.0458	0.5208	FC 2.0-4.0
					
**Array B Hsa-miR Name-Assay#**	**FC (NEVUS60/MEL60)**	**Log2(FC)**	**p value**	**FDR (BH)**	**FC Bin**

hsa-miR-7-4378130	0.3368	-1.5701	0.0014	0.1379	FC 2.0-4.0
hsa-miR-223STAR-4395209	0.0939	-3.4130	0.0045	0.1753	FC > 4
**hsa-miR-566-4380943**	8.3006	3.0532	0.0054	0.1753	FC > 4
**hsa-miR-409-3p-4395443**	0.1789	-2.4824	0.0160	0.2391	FC > 4
hsa-miR-632-4380977	1.7186	0.7812	0.0168	0.2391	FC 1.6-2.0
hsa-miR-650-4381006	0.1692	-2.5635	0.0173	0.2391	FC > 4
hsa-miR-181a-2STAR-4395428	1.7991	0.8473	0.0225	0.2391	FC 1.6-2.0
hsa-miR-432-4373280	0.0997	-3.3257	0.0233	0.2391	FC > 4
hsa-miR-571-4381016	0.3030	-1.7224	0.0237	0.2391	FC 2.0-4.0
hsa-miR-193bSTAR-4395477	3.7280	1.8984	0.0281	0.2391	FC 2.0-4.0
hsa-miR-604-4380973	0.4573	-1.1288	0.0288	0.2391	FC 2.0-4.0
hsa-miR-513-3p-4395202	3.2062	1.6809	0.0293	0.2391	FC 2.0-4.0
**hsa-miR-22STAR-4395412**	0.1556	-2.6844	0.0347	0.2495	FC > 4
hsa-miR-801-4395183	0.1982	-2.3350	0.0356	0.2495	FC > 4
hsa-miR-20aSTAR-4395548	2.5320	1.3403	0.0465	0.3040	FC 2.0-4.0

Array A: TLDA panel A (377 functionally defined microRNAs) array B: TLDA panel B (290 MicroRNAs whose function is not yet completely defined) TLDA A and B totaled 667 microRNA assays. FC: fold change; Pvalue student T test ≤ 0.05; FDR: false discovery rate; FC bin: Range of fold change. MirRs in **bold **font were found to be significantly differentially expressed between the two groups by the relative quantification (ABqPCR software-Analysis I) based method and by Class Comparison (BRB tools-Analysis II) based on absolute CT values normalized to endogenous control MammU6 (see materials and methods). N/A: not applicable.

**Table 5:** Mirs Significantly Differentially Expressed Between Pediatric And Young Adult Nevus (Nevus 30) Vs Pediatric And Young Adult Melanoma (Mel 30)

Array A Hsa-miR Name-Assay#	FC (NEVUS30/MEL30)	Log2(FC)	p value	FDR (BH)	FC Bin
hsa-miR-886-3p-4395305	0.4464	-1.1637	0.0001	0.0289	FC 2.0-4.0
**hsa-miR-449a-4373207**	0.2143	-2.2223	0.0006	0.0541	FC > 4
hsa-miR-124-4373295	0.2453	-2.0273	0.0011	0.0541	FC > 4
hsa-miR-382-4373019	0.1211	-3.0453	0.0011	0.0541	FC > 4
hsa-miR-301b-4395503	0.2264	-2.1432	0.0012	0.0541	FC > 4
hsa-miR-363-4378090	0.1417	-2.8193	0.0015	0.0577	FC > 4
**hsa-miR-22-4373079**	0.1349	-2.8895	0.0019	0.0635	FC > 4
**hsa-miR-505-4395200**	0.2482	-2.0105	0.0028	0.0749	FC > 4
**hsa-miR-135a-4373140**	0.3156	-1.6640	0.0031	0.0749	FC 2.0-4.0
hsa-miR-125b-4373148	2.0505	1.0360	0.0032	0.0749	FC 2.0-4.0
hsa-miR-518f-4395499	0.3908	-1.3554	0.0193	0.3107	FC 2.0-4.0
**hsa-miR-886-5p-4395304**	0.3436	-1.5412	0.0212	0.3107	FC 2.0-4.0
hsa-miR-517c-4373264	0.2671	-1.9043	0.0229	0.3107	FC 2.0-4.0
hsa-miR-31-4395390	0.1841	-2.4418	0.0247	0.3107	FC > 4
hsa-miR-542-3p-4378101	0.4443	-1.1704	0.0251	0.3107	FC 2.0-4.0
**hsa-miR-449b-4381011**	0.4818	-1.0536	0.0251	0.3107	FC 2.0-4.0
hsa-miR-135b-4395372	0.1699	-2.5570	0.0273	0.3107	FC > 4
hsa-miR-212-4373087	0.3822	-1.3875	0.0279	0.3107	FC 2.0-4.0
**hsa-miR-15a-4373123**	0.2598	-1.9443	0.0281	0.3107	FC 2.0-4.0
hsa-miR-362-3p-4395228	2.5474	1.3490	0.0301	0.3107	FC 2.0-4.0
hsa-miR-21-4373090	0.3731	-1.4224	0.0302	0.3107	FC 2.0-4.0
hsa-miR-134-4373299	0.4606	-1.1185	0.0305	0.3107	FC 2.0-4.0
hsa-miR-379-4373349	0.5984	-0.7408	0.0318	0.3107	FC 1.6-2.0
hsa-miR-301a-4373064	0.4202	-1.2510	0.0319	0.3107	FC 2.0-4.0
**hsa-miR-424-4373201**	0.2091	-2.2578	0.0332	0.3107	FC > 4
**hsa-miR-548b-5p-4395519**	0.5227	-0.9359	0.0382	0.3442	FC 1.6-2.0
hsa-miR-211-4373088	5.3696	2.4248	0.0400	0.3443	FC > 4
hsa-miR-494-4395476	0.2695	-1.8915	0.0412	0.3443	FC 2.0-4.0
hsa-miR-519a-4395526	0.4132	-1.2752	0.0458	0.3697	FC 2.0-4.0
					
**Array B Hsa-miR Name-Assay#**	**FC (NEVUS30/MEL30)**	**Log2(FC)**	**p value**	**FDR (BH)**	**FC Bin**

hsa-miR-650-4381006	0.1393	-2.8436	0.0000	0.0036	FC > 4
hsa-let-7iSTAR-4395283	4.3578	2.1236	0.0111	0.2768	FC > 4
hsa-miR-572-4381017	0.3278	-1.6091	0.0117	0.2768	FC 2.0-4.0
hsa-miR-135aSTAR-4395343	0.4993	-1.0021	0.0175	0.2768	FC 2.0-4.0
hsa-miR-768-3p-4395188	1.5165	0.6008	0.0181	0.2768	FC 1.2-1.6
hsa-miR-604-4380973	0.3778	-1.4043	0.0188	0.2768	FC 2.0-4.0
**hsa-miR-223STAR-4395209**	0.1451	-2.7853	0.0200	0.2768	FC > 4
hsa-miR-639-4380987	0.5274	-0.9230	0.0284	0.3442	FC 1.6-2.0
hsa-miR-214STAR-4395404	0.6008	-0.7349	0.0438	0.4602	FC 1.6-2.0
hsa-miR-409-3p-4395443	0.5134	-0.9619	0.0474	0.4602	FC 1.6-2.0

Array A: TLDA panel A (377 functionally defined microRNAs) array B: TLDA panel B (290 MicroRNAs whose function is not yet completely defined) TLDA A and B totaled 667 microRNA assays. FC: fold change; Pvalue student T test ≤ 0.05; FDR: false discovery rate; FC bin: Range of fold change. MirRs in **bold **font were found to be significantly differentially expressed between the two groups by the relative quantification (ABqPCR software-Analysis I) based method and by Class Comparison (BRB tools-Analysis II) based on absolute CT values normalized to endogenous control MammU6 (see materials and methods). N/A: not applicable.

**Table 6:** Mirs Significantly Differentially Expressed Between Adult Nevus (Nevus 60) And Young Adult/Pediatric Nevus (Nevus 30)

Array A Hsa-miR Name-Assay#	FC (NEVUS60/NEVUS30)	Log2(FC)	p value	FDR (BH)	FC Bin
None significant	N/A	N/A		N/A	
					
**Array B Hsa-miR Name-Assay#**	**FC (NEVUS60/NEVUS30)**	**Log2(FC)**	**p value**	**FDR (BH)**	**FC Bin**

hsa-miR-566-4380943	5.3288	2.4138	0.0359	0.9974	FC > 4
**hsa-miR-374aSTAR-4395236**	7.9972	2.9995	0.0371	0.9974	FC > 4

Array A: TLDA panel A (377 functionally defined microRNAs) array B: TLDA panel B (290 MicroRNAs whose function is not yet completely defined) TLDA A and B totaled 667 microRNA assays. FC: fold change; Pvalue student T test ≤ 0.05; FDR: false discovery rate; FC bin: Range of fold change. MirRs in **bold **font were found to be significantly differentially expressed between the two groups by the relative quantification (ABqPCR software-Analysis I) based method and by Class Comparison (BRB tools-Analysis II) based on absolute CT values normalized to endogenous control MammU6 (see materials and methods). N/A: not applicable.

**Table 7:** Summary Of Number Of Mirs Identified By Class Comparison Analysis I and II

Class Comparison	Array A^a^	Array B^a^	Total # of significant MiRs Array A+B Analysis I^a^	Total # of significant MiRs Array A+B Analysis II^b^	MiRs common in Analysis I and II
Mel 60 vs Mel 30	21	14	35	23	20
Nevus 60 vs Mel 60	21	15	36	35	17
Nevus 30 vs Mel 30	29	10	39	29	10
Nevus 60 vs Nevus 30	0	2	2	2	1

^a ^Number of MirRs that were found to be significantly differentially expressed at p = 0.05 level between the two groups by the relative quantification (ABqPCR software) based method. ^b^Number of MiRs identified by Class Comparison (BRB tools) based on absolute C_T _values normalized to endogenous control MammU6 (see materials and methods).

**Figure 4:**
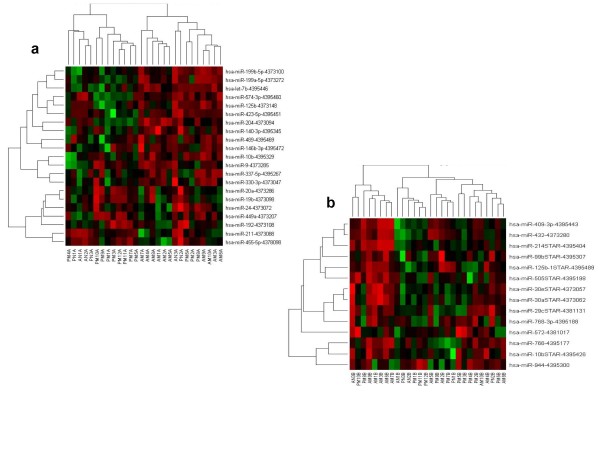
**Unsupervised Hierarchical clustering of miRs significantly differentially expressed between Mel 60 and Mel 30 groups (p ≤ 0.05); **a) TLDA A; b) TLDA B. (for MiRs statistics refer to Table 3).

**Figure 5:**
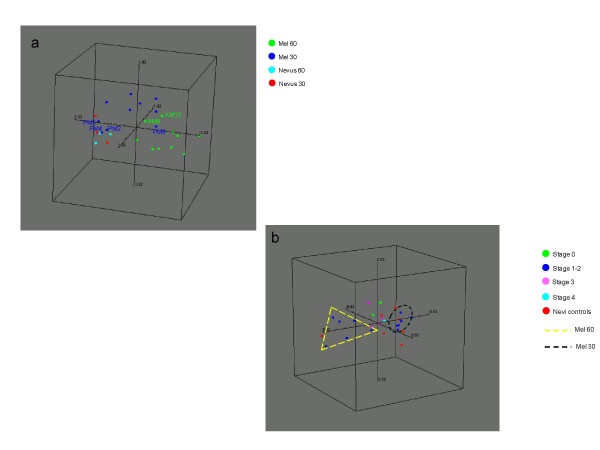
**Multidimensional scaling analysis based on 23 differentially expressed miRs between Mel 60 and Mel 30**. **a) **MSA based on the 23 miRs that by analysis II (BRB tools) differentiate Mel 60 from Mel 30 p 0.005; **b) **MSA across all stages of all samples and based on the 4 miRs (hsa-miR30a/e*, hsa-miR10b*, hsa-miR-337p) that differentiate Mel 60 stage 1-2 from Mel 30 Stage 1-2.

Differences in miR profiles between Mel 60 and Mel 30 were visualized by Hierarchical Clustering analysis (Figure 4) and by Multidimensional Scaling (MDS) analysis (Figure 5a).

Interestingly, PM8a young adult, highly atypical Spitzoid neoplasm, clustered by both methods with the adult melanoma cases.

Primary melanoma in patients greater than 60 years old (Mel 60 or AMs) was characterized by the increased expression of miRs which regulate: TLR-MyD88-NF-kappaB pathway (hsa-miR-199a), RAS/RAB22A pathway (hsa-miR-204); growth differentiation and migration (hsa-miR337), epithelial Mesenchymal Transition EMT (let-7b), hsa-miR 489, invasion and metastasis (hsa-miR-10b/10bSTAR(*), hsa-miR-30a/e*, hsa-miR-29c); regulation of cellular matrix components (hsa-miR-29c*); expressed in stem cells and still of unknown function (hsa-miR-505*); invasion and cytokinesis (hsa-miR 99b*) compared to melanoma of younger patients. In addition, as shown by Hierarchical Clustering, these miRs grouped together in signature nodes (hsa-miR -199a, let-7b, Figure 4a) (hsa-miR-30a/e*; hsa-miR-29c*, Figure 4b), indicating similar regulation and as we later confirmed from the literature, similar biological functions (see discussion-invasion and metastasis).

Interestingly the highest expression of miR-10b was observed in nodular melanoma (AM8), invasive melanomas (AM6, AM9) and desmoplastic melanoma (AM7) **(see raw CT data GEO Series accession number GSE19229 (Internet address: http://www.ncbi.nlm.nih.gov/geo/query/acc.cgi?acc=GSE19229)**. Also miR-30a* was 1 of 4 miRs significantly differentially expressed at the p-value of 0.001 between stage I-II young adult and adult melanoma (Table 8); it was 1 of the 2 miRs differentially expressed among node-positive/node-negative adults and node-positive/node-negative young adult melanomas (Table 9), and was the only miR of the 666 tested that can accurately predict classification of melanoma tissue into the young adult-pediatric vs adult groups (Tables 10 and 11).

**Table 8:** MiRs Significantly Differentially Expressed Between Stage I-II Adult Melanoma (Mel 60) And Stage I-II Young Adult-Pediatric Melanoma (Mel 30)

MiR	Parametric p-value	FDR	Permutation p-value	Geom mean of intensities in class 1	Geom mean of intensities in class 2	Fold-change
hsa-miR-30aSTAR-4373062	0.0001	0.0733	0.0022	7.7570	6.1934	1.2525
hsa-miR-30eSTAR-4373057	0.0003	0.1046	0.0022	6.8663	5.7540	1.1933
hsa-miR-10bSTAR-4395426	0.0007	0.1507	0.0022	10.5589	9.4540	1.1169
hsa-miR-337-5p-4395267	0.0009	0.1524	0.0022	17.2304	14.8781	1.1581

Stage I-II Adult melanoma were compared with stage I-II pediatric melanoma by Two-sample T-test on the global data set of 666 miRs C_T _values normalized to MammU6 endogenous control (see analysis II). Class 1: Mel 30 Stage I-II; Class 2: Mel 60 Stage I-II. Exact permutation p-values for significant genes were computed based on 462 available permutations. Nominal significance level of each univariate test: 0.001. Global test: probability of getting at least 4 genes significant by chance (at the 0.001 level) if there are no real differences between the classes: 0.02597.

**Table 9:** Mirs Differentially Expressed Between Node Positive And Node Negative Adult (Mel 60) And Young Adult-Pediatric (Mel 30)

MiR	Parametric p-value	FDR	Permutation p-value	Geom mean of intensities in class 1	Geom mean of intensities in class 2	Geom mean of intensities in class 3	Geom mean of intensities in class 4
hsa-miR-204-4373094	0.00004	0.02784	< 1e-07	15.74986	11.70222	14.82001	6.94659
hsa-miR-30aSTAR-4373062	0.00035	0.11658	0.00010	7.67985	6.27768	7.25131	6.95430

The univariate F-test at the nominal significance level of 0.001 was performed among 4 classes: Class 1: Node-negative-Mel 30; Class 2: Node-negative-Mel 60; Class 3: Node-positive-Mel30; Class 4: Node-positive-Mel60. Permutation p-values for significant MiRs were computed based on 10000 random permutations. The Global test: probability of getting at least 2 genes significant by chance (at the 0.001 level) if there are no real differences between the classes was 0.137.

**Table 10:** Class Prediction Analysis: Young Adult-Pediatric (Mel 30) vs Adult Melanoma (Mel 60)

Parametric p-value	t-value	% CV support	Geom mean of intensities in class 1	Geom mean of intensities in class 2	Fold-change	MiR
0.00008	5.05700	100.00000	7.60029	6.47345	1.17407	hsa-miR-30aSTAR-4373062

Class prediction analysis was computed using BRB tools between Class 1: Mel 30 (10 specimens) and Class 2: Mel 60 (10 specimens) across the global data set of 666 MammU6 normalized MiRs (Analysis II). MiRs significantly different between the classes at 0.001 significance level were used for class prediction.

**Table 11:** Performance Of Classification Methods used for Class Prediction Analysis

Performance of the Compound Covariate Predictor Classifier:				
**Class**	**Sensitivity**	**Specificity**	**PPV**	**NPV**
**Mel 30**	0.8	0.9	0.889	0.818
**Mel 60**	0.9	0.8	0.818	0.889

**Performance of the Diagonal Linear Discriminant Analysis Classifier:**				

**Class**	**Sensitivity**	**Specificity**	**PPV**	**NPV**
**Mel 30**	0.8	0.9	0.889	0.818
**Mel 60**	0.9	0.8	0.818	0.889

**Performance of the 1-Nearest Neighbor Classifier:**				

**Class**	**Sensitivity**	**Specificity**	**PPV**	**NPV**
**Mel 30**	0.8	0.8	0.8	0.8
**Mel 60**	0.8	0.8	0.8	0.8

**Performance of the 3-Nearest Neighbor Classifier:**				

**Class**	**Sensitivity**	**Specificity**	**PPV**	**NPV**
**Mel 30**	0.7	0.9	0.875	0.75
**Mel 60**	0.9	0.7	0.75	0.875

**Performance of the Nearest Centroid Classifier:**				

**Class**	**Sensitivity**	**Specificity**	**PPV**	**NPV**
**Mel 30**	0.8	0.9	0.889	0.818
**Mel 60**	0.9	0.8	0.818	0.889

**Performance of the Support Vector Machine Classifier:**				

**Class**	**Sensitivity**	**Specificity**	**PPV**	**NPV**
**Mel 30**	0	0	0	0
**Mel 60**	0	0	0	0

**Performance of the Bayesian Compound Covariate Classifier:**				

**Class**	**Sensitivity**	**Specificity**	**PPV**	**NPV**
**Mel 30**	0.7	0.5	0.583	0.625
**Mel 60**	0.5	0.7	0.625	0.583

The performance of classification methods used for class prediction analysis in Table 10 was conducted as follows: the Leave-one-out cross-validation method was used to compute mis-classification rate. Based on 100 random permutations, compound covariate predictor p-value = 0.04, diagonal linear discriminant analysis classifier p-value = 0.04, 1-nearest neighbor classifier p-value = 0.02, 3-nearest neighbors classifier p-value = 0.03, nearest centroid classifier p-value = 0.04, support vector machines classifier p-value = 0.72, Bayesian compound covariate classifier p-value = 0.05. For each classification method and each class: Sensitivity = the probability for a class A sample to be correctly predicted as class A, Specificity = probability for a non class A sample to be correctly predicted as non-A, PPV = probability that a sample predicted as class A actually belongs to class A, NPV = probability that a sample predicted as non class A actually does not belong to class A.

T-values used for the (Bayesian) compound covariate predictor were truncated at abs(t) = 10 level. Equal class prevalence was used in the Bayesian compound covariate predictor. Threshold of predicted probability for a sample being predicted to a class from the Bayesian compound covariate predictor was 0.8. % CV support proportion of the cross-validation loops that contained each MiR in the classifiers. T value = ratio of the estimate divided by the standard error.

On the contrary, other well known miRs were found to be downregulated in the older age group melanomas compared to younger age group melanomas: hsa-miR-211; hsa-miR 455-5p, hsa-miR-24; hsa-miR944. It is interesting that expression of miR 211 is dramatically downregulated in primary melanomas compared to nevi control and decreases with increasing age (Table 3, 4 and Figure 4).

Primary melanoma in young adult patients (Table 3, 5 and Figure 4) was characterized by the increased expression of hsa-miR 449 a (Mel 60< Mel 30> Nevus 30) and decreased expression of hsa-miR146b (Mel 60> Nevus 60 and >Mel 30) hsa-miR 214* (Mel 60>Mel 30 Mel 30 > Nevus 30).

Among the miRs expressed at higher levels in the control nevi compared to adult or young adult melanoma was hsa-miR 574-3p (Nevus 60> Mel 60> Mel 30).

Only 2 miRs distinguished adult from young adult-pediatric nevi, hsa-miR374a* and has-miR-566 (Table 6). The latter miR was expressed at 8-fold higher levels in the adult nevi than in the adult melanoma (Table 4).

To analyze similarities and dissimilarities between primary melanomas and nevi in miR profiles relative to clinical and pathological diagnosis, we performed a class comparison analysis by two-sample t-test between Stage I-II adult and young adult-pediatric melanoma. Four miRs: hsa-miR 30 a*/e*, hsa-miR -10b*, hsa-miR- 337-5p were found to be significantly differentially expressed between the two groups, composed of 6 patients each (Tables 2, 8). Multidimensional Scaling Analysis was utilized to visualize the striking miR profiling that clearly segregated adult from young adult cases and nevi controls (Figure 5b).

To investigate whether nodal involvement (related to age) could be correlated with the expression of a specific set of miRs, we conducted a univariate F-test among four groups consisting of node positive adult, node negative adult, node positive young adult-pediatric, node negative young adult-pediatric.

Two miRs were found to be significantly differentially expressed among the 4 classes: hsa-miR-204 and hsa-miR-30a* (Table 9).

In order to explore the possibility that a set of miRs could aid in the classification of young adults vs. adult melanoma, Class Prediction analysis was computed using BRB ArrayTools between Mel 30 (10 specimens) and Mel 60 (10 specimens) across the global data set of 666 MammU6 normalized miRs (Analysis II). MiRs that significantly differed between the classes at 0.001 significance level were used for class prediction classification. Hsa-miR 30a* (Tables 10 and 11) was found to be a potential candidate predictor.

## Discussion

A limited number of miRs has been discovered expressed in melanoma and correlated with dysregulated pathways of growth and metastasis [15,32-38]**(miR modulated in melanoma -Melanoma Molecular Map project http://www.mmmp.org/MMMP/)**.

Only two studies to date have addressed the importance of characterizing melanoma tissue (as opposed to cell lines) by miR profiling. Schultz et al. reported on a new regulatory mechanism of early melanoma development [35]. These authors analyzed 157 miRs in laser-microdissected tissues from benign melanocytic nevi and primary malignant melanomas using quantitative real-time PCR and found 72 microRNAs differentially expressed between melanoma and nevus tissue. Members of the let-7 family of microRNAs were significantly downregulated in primary melanomas as compared with benign nevi, suggesting a possible role of these molecules as tumor suppressors in melanoma. Let-7b inhibited cell cycle progression and anchorage-independent growth of melanoma cells.

The second study [36] investigated the value of miRNA expression patterns in predicting metastatic risk in uveal melanoma, previously described to consist of two distinct subtypes: high- and low-risk of metastatic death. After screening 470 human miRs, Worley et al. found that miR-let-7b and miR-199 were the most significant predictors for the two classes.

Our miRNA profiling of FFPE-primary melanomas obtained from older adults and pediatric or young adult patients in relation to age-matched nevus controls represents the first intergenerational study to analyze expression of 666 miR in primary melanomas and control nevi. Although we acknowledge that our findings need to be further validated on an independent set of adult and young adult/pediatric fresh frozen specimens, the descriptive mining analysis we conducted (summarized in **Additional file **2) reveals the specific gene expression regulation of the melanoma tumor types in the two groups of patients, which are separated by at least 30 years in age. We report several miRs with expression profiles paralleling those described in the literature for melanoma and other cancers (ovarian, breast, lungs, pancreas) and miRs with expression modulated in the opposite direction. This is not surprising since, as Nicoloso et al.,[20], miRs are tissue- and tumor-specific; there seems to be a tumor-specific pattern of miR gene modulation [13].

Hierarchical Clustering and MDS analysis substantiated the clinical observations that melanoma in the older population studied here differs significantly from the melanoma of younger patients. It is of particular interest that the only young adult female lesion classified as an atypical Spitzoid neoplasm (PM8) clustered with the adult melanoma cases. This finding provides us with additional information about the the still-puzzling and complex pathological diagnosis of Spitzoid neoplasms [39-42].

Barnhill et al. report on the need to perform a systematic and rigorous evaluation of Spitzoid lesions utilizing all histopathological, clinical, and ancillary information [43] Although our report includes only one such lesion, it suggests that miR profiling of Spitzoid lesions may provide that ancillary molecular data, which could be of aid in the formulation of the pathological evaluation and in risk assessment and stratification.

Primary melanoma in patients older than 60 was characterized, in particular, by the increased expression of hsa-miR-204, hsa-miR-199a, hsa-miR337, let-7b, hsa-miR-489, hsa-miR-10b/10b*; hsa-miR-30a/e*; hsa-miR-29c*; hsa-miR-505*; and hsa-miR 99b* compared to melanoma of younger patients (<30), indicating similar regulation, and as we later confirmed from the literature, similar biological functions (see discussion-invasion and metastasis).

MiR-204 was significantly (34 fold) upregulated in older adult versus younger adult/pediatric melanomas. This miR is normally expressed in the choroid plexus, retinal pigment epithelium, and ciliary body [44]. Its expression is reported in insulinomas and directly correlates with immunohistochemical expression of insulin [45]. In acute myeloid leukemia, miR-204 targets HOXA10 and MEIS1, two members of the homeobox family of transcription factors involved in leukemia development [46]. Wu et al. reported that **miR-204**, miR-99b, and miR-193b were greatly downregulated in adenocarcinoma tissues while miR-205, **miR-449**, and miR-429 were greatly enriched [47].

Comparative genomic hybridization (CGH) studies of DNA copy number abnormalities in genomic regions containing known miRNA genes showed that miR-204 is downregulated in a minority of melanoma cell lines [48]. Schultz et al. reported down-regulation of miR-204 in primary malignant melanomas compared to benign nevi [35]. In contrast to this data, we are the first to report that miR-204 expression is greatly increased in primary melanomas of patients older than 60 compared to melanomas of younger adults and pediatric patients younger than 30. The biological significance of this finding in melanoma represents a compelling subject for future investigation considering that, in addition to the targets cited above (HOXA10 and MEIS1), another predicted target of miR-204 is RAB22A, a member of the RAS oncogene family, which is involved in the trafficking from endosomes to the Golgi apparatus **(Internet address: http://pictar.mdc-berlin.de/cgi-bin/PicTar_vertebrate.cgi algorithm for the identification of miR target)**. RAB22A was found to reside in regions of chromosomal breakpoints and has altered/increased expression in melanoma [49]**(Additional file **3).

**Hsa-miR-199a **was more than 4 fold upregulated in adult melanomas (>60 years) compared to young melanomas (<30 years). This miR may not only be a critical biomarker of differentiation between adult and young adult melanomas but may also play an important role in the tumor microenvironment and provide a potential target for tumor treatment. Chen et al. recently identified hsa-miR-199a as a regulator of IKKbeta expression [50]. High miR-199a expression leads to inhibition of IKKbeta, and these authors showed that IKKbeta is a major factor promoting a functional TLR-MyD88-NF-kappaB pathway, which is associated with the capacity to constitutively secrete proinflammatory/protumor cytokines in ovarian cancer, whereby promoting tumor progression and chemoresistance. Chen et al. report that Type I epithelial ovarian cancer (EOC) cells have high levels of IKKb expression due to low hsa-miR-199a; therefore, when stimulated, nuclear factor-kB (NF-kB) activation leads to cytokine production, cell proliferation and induction of antiapoptotic proteins. In Type II EOC, cell expression of IKKb is low due to high hsa-miR-199a expression, which blocks the TLR4-MyD88-NF-kB pathway response to ligands and inhibits cytokine production, resulting in chemosensitivity. IKKb is highly active in many other different types of cancer including melanoma [51].

It is possible that melanomas in older patients (>60) with high levels of hsa-miR-199a are similar to Type II EOC, have low NFKB expression levels and a less inflammatory microenvironment. By contrast, melanoma in the younger age group would appear similar to Type I EOC cells, with high levels of IKKb expression due to low hsa-miR-199a that, when stimulated by nuclear factor-kB (NF-kB) activation, would lead to cytokine production, cell proliferation and induction of anti-apoptotic proteins as a result of the expression of an active IKKbeta pathway. It remains to be evaluated and it is the object of our future studies, whether the tumor inflammatory cytokine profile in adult melanomas is downregulated with respect to young adult-pediatric melanomas as a consequence of differential NFKB activation.

There is clear evidence that lymph node metastases are more prevalent among younger patients with melanoma compared to the adult population, suggesting that melanoma cells in the young are more prone to progression and to subsequent invasion and metastasis [52] Sondak et al. reviewed 419 patients who underwent sentinel lymph node (SLN) biopsy for melanoma from a prospectively collected melanoma database and reported that high mitotic rate and younger age are predictors of SLN positivity [53].

Interestingly, the finding that high miR-199a expression leads to inhibition of IKKbeta and downregulation of the TLR-MyD88-NF-kappaB pathway is consistent with other lines of evidence that suggests that miR-199a/a* is indeed a putative tumor suppressor. Expression of miR-199a/a* is silenced in all proliferating cell lines tested except fibroblasts; introduction of miR-199a/a* caused apoptosis in cancer cells; miR-199a* down-regulates MET proto-oncogene and also down-regulates ERK2, an effector downstream of MET (Additional file 2 and 3) [54].

The observation that hsa-miR-337-5p is differentially upregulated in melanomas developing in older compared with younger patients is a novel finding. Not much is known to date in regard to the role of **hsa-miR-337-5p **in cancer. It appears that this miR may be involved in regulation of cell growth, differentiation and migration. Hussein et al. reported that over-expression of Lyn tyrosine kinase, a marker of leukemic cell growth in B-CLL, was associated with a significant down-regulation of microRNA-337-5p [55]. Palmieri et al. found that miR-337 was upregulated in osteoblast-promoting bone formation and in turn regulated the expression of genes related to receptors (growth hormone releasing hormone receptor, GHRHR) and extracellular matrix proteins (cartilage oligomeric matrix protein, COMP) [56].

The upregulation of miR-let-7b in the adult compared to the pediatric and young adult group is intriguing, in view of the finding of Schultz whereby forced overexpression of let-7b in melanoma cells in vitro downregulates the expression of cyclin-D1, D3, A, and cyclin dependent kinase (CDK4), all of which have been described to play a role in melanoma development [35] (Additional file 3). Consistent with its down-modulating effects on cell cycle regulators, overexpression of let-7b inhibited cell cycle progression and anchorage-independent growth of melanoma cells.

Furthermore, Lee at al.,[57] showed that there is a direct linkage between let-7b and the high-mobility group protein and oncogene (HMGA2). HMGA2 is a non-histone chromatin factor that is primarily expressed in undifferentiated tissues, tumors of mesenchymal origin and lung cancer. In pancreatic cancer cells, this protein maintains Epithelial Mesenchymal Transition (EMT) [58] Let-7b negatively regulates HMGA2 and, by repressing this oncogenic target, acts as growth suppressor [57].

MiR let-7b is expressed 2-fold higher in the melanoma of older patients (Mel 60 group) compared to younger patients (Mel30) we studied, which is of interest considering the function of this inhibitor of cell cycle progression and EMT (Additional file 2). This is then similar to the case we made for miR-199a. The fact that lymph node metastases are more prevalent in young people with melanoma compared to adults [52] suggests that melanoma cells in the young are more prone to EMT progression and subsequent invasion and metastasis, compared with melanoma cells of older populations. Expression of cyclins-D1, D3 A and CDK4, as well as HMGA2 in adult and young adult-pediatric melanomas represents a central and future focus for our comparison of transgenerational melanoma specimens.

We found statistically significant changes in the same 2 miRs, let-7b and with miR-199a, previously reported by Worley et al. as important biomarkers of melanoma. Expression of miR-let-7b and miR-199a differentiate ocular melanoma of high- and low risk for metastasis [36]. It is notable that in ocular melanoma the upregulation of these two miRs denoted high metastatic potential while in cutaneous melanoma upregulation was linked to inhibition of growth and EMT.

The significance of differential upregulation of hsa-miR-489 is elusive. This miR is essential for the regulation of osteogenesis by down-regulating differentiation of mesenchymal stem cells [59].

The two-fold upregulation of hsa-miR-10b/10b(*) expression in adult melanoma, compounded with the observation that expression of this miR is significantly differentially expressed between adult and young patients with stage I-II melanoma (Table 8) is of particular importance, because miR-10b and, its less predominant form miR-10b*, have been reported to be upregulated in prostate cancer [31,60] pancreatic cancer [61] ovarian cancer [62] glioblastoma [63] metastatic breast cancer [64] chronic lymphocytic leukemia [65] and melanoma cell lines [48].

More specifically, miR-10b appears to be a key oncomiR associated with metastasis: it is induced by Twist and proceeds to inhibit translation of the messenger RNA encoding homeobox D10, which results in increased expression of the well-characterized pro-metastatic gene RHOC. Overexpression of miR-10b in otherwise non-metastatic breast tumors initiates robust invasion and metastasis. Thus miR-10b positively regulates cell migration and invasion, and its high expression correlates with clinical progression in breast cancer [64]. Furthermore, Hutchison et al. recently demonstrated that RhoC has a distinct and specific function in the process of epithelial-to-mesenchymal transition (EMT) in renal proximal tubular cells. RhoC is the isoform solely responsible for stress fiber formation, and inhibiting its expression reduces EMT-induced migration by 50% [66].

The specimens with highest expression of miR-10b were an adult nodular melanoma (AM8, Stage 1B), 2 invasive thinner adult melanomas (AM6, AM9 Stage IA) and a deeper desmoplastic melanoma (AM7, Stage IIB). These observations suggests that miR-10 is a candidate biomarker for metastatic potential of localized early stage melanoma (Stage I-II). While our study included diverse morphotypes, a larger study to evaluate morphotypes is required to validate the predictive value of this molecule.

Similar to hsa-miR10b, hsa-miR-30a*/e*, which was upregulated in the melanoma of older adults compared to the young, is a biomarker of metastasis in liver cancer [67]. MiR-30a is part of a 20-miRNA metastasis signature that may distinguish primary hepatocellular carcinoma (HCC) tissues with venous metastases from metastasis-free solitary tumors with 10-fold cross-validation. The 20-miRNA tumor signature including miR-30a was validated as a significant, independent predictor of survival and relapse [67].

It is not surprising that among miR-30a-predicted targets are molecules directly related to cell proliferation and inflammation: mitogen activated protein kinase 5 (MAP3K5), the RAS related protein RAB32 and the suppressor of cytokine signaling, SOC1 (Internet address: http://www.ebi.ac.uk/enright-srv/microcosm/cgi-bin/targets/v5/search.pl

Important in the characterization of primary melanoma and its metastatic potential, we report that miR-30a* is 1 of 4 miRs significantly differentially expressed at the p-value of 0.001 between stage I and II young adult and adult melanomas (Table 8); it is 1 of the 2 miRs differentially expressed among node-positive and node-negative adult melanomas as well as between node-positive and node-negative young adult melanomas (Table 9); and it is the only miR out of 666 tested that can accurately predict classification of melanoma tissue into the young adult-pediatric vs. adult groups (Tables 10 and 11).

Although hsa-miR-29c* was found to be down-regulated in nasopharyngeal carcinoma (NPC), ovarian, lymphoma and other cancers [62,68,69], we report that this miR was 2 fold higher in adult melanomas compared to young adult-pediatric melanomas. We hypothesize that that miR-29c could have an important regulatory function in the stroma surrounding the tumor microenvironment, given the critical cancer role of its predicted targets (Internet address: http://www.ebi.ac.uk/enright-srv/microcosm/cgi-bin/targets/v5/search.pl, http://pictar.mdc-berlin.de/cgi-bin/PicTar_vertebrate.cgi) encoding extracellular matrix proteins associated with cellular matrix, migration and metastasis, several collagen alpha-chain precursors, disintegrin and metalloproteinase precursors (ADAMS), and TNF related proteins (Additional file 2). Further investigations focused on the regulatory mechanism of these predicted targets are undoubtedly necessary to support this hypothesis.

Several of the miRs we report as upregulated in this study among adult melanomas have recently been described collectively as under-expressed in renal acute rejection biopsies compared to normal allograft biopsies [70]**(let-7c, miR-10b, miR-30a-3p, miR30e-3p)**. This makes sense biologically, that a group of miR-regulators of cell growth, proliferation, invasion, and survival would be upregulated in a persisting, progressing tumor and downregulated in tissue being rejected. Furthermore, our current observations are concordant with the similarity in mRNA transcripts expression between renal allograft rejection and melanoma that we previously described [71].

We acknowledge the necessity of testing the effect of silencing these miRs and assessing their modulation in a setting of mixed responses, in areas of ongoing tumor rejection vs. tumor progression (by FNA) [71]. These experiments would help to establish whether this group of miRs does, in fact, constitute candidates for targeted therapies.

Hsa-miR-505*; is a relatively newly discovered miR that has been recently found to be among the 10% more significantly differentially expressed in undifferentiated human Embryonic Stem Cells (hESC) [72]. We are the first to report the modulation of this miR in the context of melanoma. It is possible that the upregulation of this miR in the adult melanoma indicates the activation of cancer stem cells, but this hypothesis would need to be tested.

Hsa miR 99b* along with miR-10, miR-125b and miR-30, are upregulated in adult compared to young age melanomas. This observation overlaps with the findings of Prueitt et al., [60] in prostate cancer. The authors showed that these same microRNAs were greater than 2 fold upregulated in prostate cancer with perineural invasion (PNI), the dominant pathway for local invasion in prostate cancer vs. prostate cancer without PNI. Predicted PIC Tar targets for miR-99b include calmodulin 2 (CALM2), which mediates the control of several protein kinases and phosphatases and is involved in the pathway that regulate the centrosome cycle and progression through cytokinesis.

Among the miRs that we found were downregulated in older age melanomas compared to younger melanoma, were hsa-miR-211, hsa-miR-455-5p, hsa-miR-24 and hsa-miR944. The expression of hsa-miR-211 is dramatically downregulated in primary melanoma compared to nevi control and decreases with increasing age (Table 3, 4 and Figure 4). Very little is known about the function and targets of this miR. Our observation is in contrast to the 1.4 fold upregulation of this miR in primary melanoma, compared to benign nevi reported by Schultz, et al., [35]. It is also contrary to the upregulation of miR-211 in oral carcinoma, which was associated with the most advanced nodal metastasis, vascular invasion, and poor prognosis [73].

It is very intriguing that among the miRbase predicted target genes **(Internet address: http://www.ebi.ac.uk/enright-srv/microcosm/cgi-bin/targets/v5/search.pl) **of miR-211 is the CC-Chemokine receptor 10 (CCR10) (Additional file 2) which is expressed in melanocytes, dermal fibroblasts, dermal microvascular endothelial cells, T-cells, and skin-derived Langerhans cells. CCR10 binds the inflammatory chemokines MCP-1, MCP-3 MCP-4, RANTES and CTACK-CCL27 which selectively attracts circulating memory T-cells that specifically express the cutaneous lymphocyte-associated antigen CLA **(internet address: http://www.copewithcytokines.de/cope.cgi?key=CCR10)**

The progressive age dependent-down-regulation of miR-211 observed in melanoma, compared to a benign nevus microenvironment, may therefore underlie the importance of further studying what appears to be a master immuno-regulatory role of this miR in the melanoma tumor microenvironment, EMT and invasion. As discussed adult melanomas invasive capacity maybe related more to the de-regulated activity of miRs impacting on EMT, stromal components, cell cycle and growth differentiation and on the reduction of inflammatory pathways (upregulated miR-199a, miR-let 7b, miR-10b, miR30a, miR99b); whereas young melanomas seems to be driven by regulatory molecules more targeted at increasing inflammation in the tumor microenviroment (low miR-199, miR-211, miR-944).

Co-downregulation of miR-455, -24 and -944 in adult melanoma, compared to young adults, is certainly of biological significance since these miRs are involved in metabolic (miR-455), and cell repair mechanisms (miR-24), as well as inflammation-immunity-differentiation and cell growth (miR-944). Hsa-miR-455-5p-increasing expression correlates with the differentiation process of brown adipocytes, while decreased expression of miR-455 occurs in muscle tissue where large changes in metabolic capacity take place [74] MiR-24-mediated downregulation of H2AX suppresses DNA repair in terminally differentiated blood cells [75]. Hsa-miR944 is a novel miR [76] that has among its predicted targets **(internet address: http://www.ebi.ac.uk/enright-srv/microcosm/cgi-bin/targets/v5/search.pl)**, the C-Rel proto-oncogene, one of the five transactivator members of the REL/NFkb family [77], suggesting a role for miR-944 in the regulation of NFKb.

It is conceivable that these miRs, including miR-24, would be in the group of candidate miR biomarkers previously discussed, that partially explain the ability of the young adult melanomas to metastasize more frequently to the lymph nodes (low miR-199). Our observations, corroborated by similar findings in other cancers, suggest that adult melanomas may rely on different pathways of invasion than young adult melanomas.

Regarding the characterization of nevus tissue, we are the first to report that only 2 miRs distinguished adult from young adult-pediatric nevi: hsa-miR374a* and has-miR-566. The MiR-374a* predicted targets FL cytokine receptor precursor (FLT3); BRCA2 and CDKN1A-interacting protein (BCCIP); CD9 antigen (p24, Leukocyte antigen MIC3, Motility-related protein, MRP-1)**(Internet address: http://www.ebi.ac.uk/enright-srv/microcosm/cgi-bin/targets/v5/search.pl)**, seem to suggest a possible regulatory role of this miR in immune regulation, DNA repair and cell cycle.

The expression of hsa-miR-566 was 8 fold higher in adult nevi compared to adult melanomas and 5 fold higher compared to the young adult nevi. While to our knowledge, the regulatory function of this miR has not yet been elucidated, our observation suggests that marked upregulation of hsa-miR 566 expression level maybe considered a distinguishing feature of normal nevus tissue compared to melanoma and dysregulation/downregulation of miR 566 expression could be considered a putative marker of the malignant melanoma phenotype in advanced age.

Particularly puzzling was the expression of hsa-miR-449a across the miRnome of the adult and young adult/pediatric melanomas and nevi. Hsa-miR-449a downregulation in adult melanomas is consistent with the downregulation of miR-449a found in prostate cancer tissues and the recent discovery that histone deacetylase 1 (HDAC-1) is a target of miR-499 [78]. HDAC is frequently over-expressed in a broad range of cancer types where it alters cellular epigenetic programming to promote cell proliferation and survival. High miR-499 expression allows repression of HDAC expression and consequent inhibition of cell proliferation, while downregulation of miR-499 promotes cell growth. It remains unexplained why miR 499 is downregulated in young adult nevi compared to young adult melanomas.

Finally, hsa-miR-146b and hsa-miR-214* were both found to be upregulated in adult compared to young adult melanomas and down-regulated in the age-matched nevi tissue. Hsa-miR-146b upregulation in melanoma confirms the data of Igoucheva et al.,[32] that reports upregulation of miR-146b with vertical growth pattern and metastatic melanoma compared to normal melanocytes.

The expression of miR-214* was similarly upregulated in adult melanomas compared to young melanomas, but downregulated in young adult nevi compared to young adult melanomas. This miR has been reported to be upregulated in lung, pancreatic, gastric cancer and down-regulated in hepatocellular carcinoma **[Internet address: http://www.mir2disease.org/]**

Interestingly while there are no reports to our knowledge on the expression of miR-214 in melanoma, miR-214 is a miR predicted to target the tumor suppressor gene PTEN, which is absent or significantly reduced in melanoma (Additional file 3).

## Conclusions

Our analysis of the miRnome of pediatric and young adult melanomas in relation to older adult melanomas provides a new basis for characterization of melanoma at the extremes of age. Our findings, although preliminary and obtained from a relatively small number of FFPE specimens, support the notion that the differential biology of this disease at the extremes of age is driven, in part, by deregulation of microRNA expression and by fine tuning of miRs that are already known to regulate cell cycle, inflammation, EMT/stroma and more specifically genes known to be altered in melanoma. Furthermore, our analysis reveals that miR expression differences create unique patterns of frequently affected biological processes that clearly distinguish old age from young age melanomas.

## Competing interests

The authors declare that they have no competing interests.

## Authors' contributions

DMJ was project co-PI and reference pathologist, selected FFPE of adult and pediatric melanoma and control lesions, reviewed the manuscript. UNMR was responsible for original collection of melanoma specimens, reference pathologist for primary evaluation of adult and pediatric melanoma cases, provided advice and assisted with the writing of the manuscript. LK assisted with specimen retrieval and selection from Health Sciences Tissue Bank, isolated RNA, conducted TLDA assays and organized raw data, equal contribution as first author. JSS carried out microRNA analysis and assisted in interpreting the data (using ABqPCR software). LMD assisted in the retrieval of the FFPE specimens, selection of cases and editing of the manuscript. JMK performed the original clinical evaluation of the patients from whom the archived lesions were obtained, provided advice on the project and manuscript. MCP was project PI, designed the study, carried out microRNA analysis (using BRB tools), and wrote the manuscript.

All authors read and approved the final manuscript.

## Supplementary Material

Additional file 1 **Supplemental file**. Study Schema Click here for file

Additional file 2 **Supplemental table**. Summary Of MiRs Characteristic Of Adult And Young Adult-Pediatric Melanoma And Their Predicted Gene Targets Click here for file

Additional file 3 **Supplemental table**. Genes deregulated in melanoma and miRs predicted to target these genes [79] Click here for file
