# Supplementary material for: Microrna profiling analysis of differences between the melanoma of young adults and older adults
Source: J Transl Med. 2010 Mar 19;8:27. doi: 10.1186/1479-5876-8-27 (PMC2855523; doi:10.1186/1479-5876-8-27)
Supplement: Additional file 1 — Supplemental file. Study Schema [file 1479-5876-8-27-S1.PPT]

## Slide 1
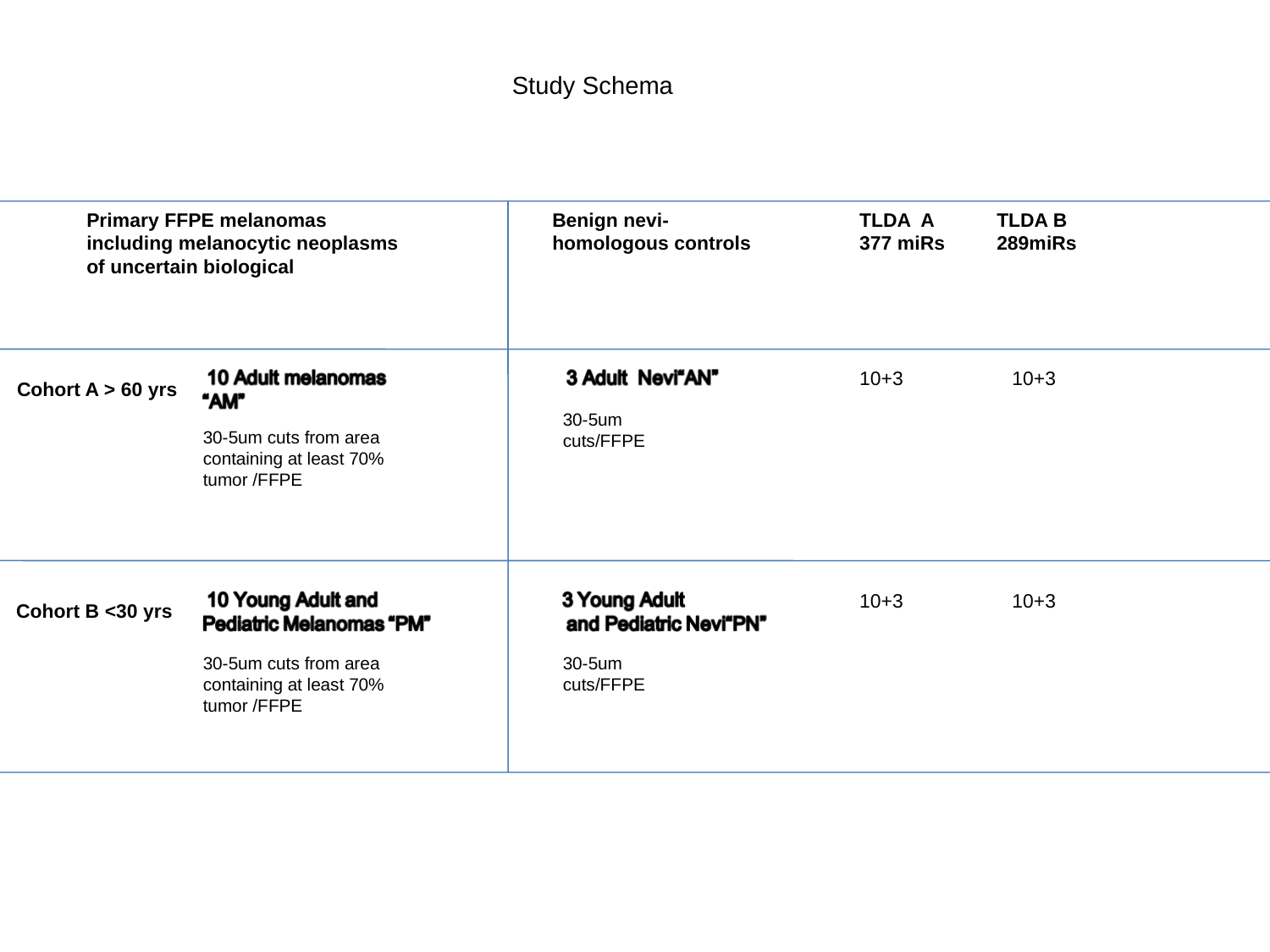

Study Schema
Primary FFPE melanomas including melanocytic neoplasms of uncertain biological
Benign nevi-
homologous controls
TLDA A
377 miRs
TLDA B
289miRs
10+3
10+3
Cohort A > 60 yrs
30-5um cuts/FFPE
30-5um cuts from area containing at least 70% tumor /FFPE
10+3
10+3
Cohort B <30 yrs
30-5um cuts from area containing at least 70% tumor /FFPE
30-5um cuts/FFPE
