# Supplementary material for: Microrna profiling analysis of differences between the melanoma of young adults and older adults
Source: J Transl Med. 2010 Mar 19;8:27. doi: 10.1186/1479-5876-8-27 (PMC2855523; doi:10.1186/1479-5876-8-27)
Supplement: Additional file 2 — Supplemental table. Summary Of MiRs Characteristic Of Adult And Young Adult-Pediatric Melanoma And Their Predicted Gene Targets [file 1479-5876-8-27-S2.DOC]

**Additional file 2. Summary Of MiRs Characteristic Of Adult And Young Adult-Pediatric Melanoma And Their Predicted Gene Targets**

| **MiR** | **Chromosomal location** | **Expression in adult** | **Expression in young adult/pediatric** | **Known Targets (Entrez,PubMed)** | **Predicted target related to cancer (MiRbase**  **/PicTar)** | **Summary of functions** | **Comment melanoma cases in this report and melanoma literature** | **Previously reported in Melanoma vs *this report ( Jukic et al.,)*** |
| --- | --- | --- | --- | --- | --- | --- | --- | --- |
| hsa-mir-204 | Chr: 9; Location: 9q21.11 | higher | lower | HOXA10 and MEIS1 | RAB22A  BCL2 | Involved in AML development [46], deregulated in insulinomas [45], adenocarcinoma [47]  Exact biological function in Melanoma not yet defined | RAB22A (RAS family) target has been found to reside in regions of chromosomal breakpoints (20q13) and have altered/increased expression in melanoma [49]  *Deregulation of miR 204 maybe due to the fact hat Chr 9 is a site of Consistent translocation in melanoma:  der(9)t(9;22)(p21;q11) [49] | Downregulated in Mel cell lines [48]  *miR-204 expression is greatly increased in primary melanomas( FFPE) of patients > 60 years compared to < 30 years patients* |
| hsa-mir-199a | Chr: 19; Location: 19p13.2 | higher | lower | IKKbeta,TLR4–MyD88–NF-kB pathway MET proto-oncogene, ERK2 |  | Inhibitor of IKKb [50], down-regulates MET proto-oncogene and ERK2 [54].  Classified as tumor suppressor reduces inflammation | MET and ERK are disregulated in Melanoma (seeTable 11) | Downregulated in Mel cell lines [48]  Downregulated in ovarian cancer [50]  *miR-199a expression is greatly increased in primary melanomas( FFPE) of patients > 60 years compared to < 30 years patients* |
| hsa-miR-337-5p | Chr14 location:14q32.31 | higher | lower | COMP |  | Upregulation in osteoblast promotes bone formation and regulates extracellular matrix proteins COMP |  | *miR-337 expression is increased in primary melanomas( FFPE) of patients > 60 years compared to < 30 years patients* |
| Let-7b | Chr22; Location: 22q13.31 | higher | lower | HMGA2,  cyclin-D1, D3, A,  CDK4 |  | Regulation of developmental timing and cellular proliferation; Inhibition of cell cycle progression and EMT | let-7c, miR-10b, miR-30a-3p, miR30e-3p  collectively underexpressed in a rejection microenvironement (renal acute rejection biopsies )  but upregulated in our study in adult melanomas | Upregulated in Mel cell lines [48]  *Let-7b expression is increased in primary melanomas( FFPE) of patients > 60 years compared to < 30 years patients* |
| hsa-miR-10b | Chr 2; Location: 2q31.1 | higher | lower | Homeobox D10-RHOC prometastatic gene (RhoC is the RHO isoform solely responsible for stress fiber formation and inhibiting its expression reduces EMT-induced migration by 50% [66] |  | marker of progression, metastasis breast cancer, EMT | highest in adult nodular melanoma ( case AM8,Stage 1B) invasive melanomas (cases AM6, AM9 stage 1A) and desmoplastic melanoma ( case AM7, Stage 2 B)  let-7c, miR-10b, miR-30a-3p, miR30e-3p  collectively under-expressed in a rejection microenvironement (renal acute rejection biopsies ) [70]  but upregulated in our study in adult melanomas |  |
| hsa-mir-30a | Chr 6; Location: 6q13 | higher | lower |  | MAP3K5 RAB32  SOC1 | marker of metastasis in lung cancer  and in acute renal allograft rejection | SOC1 target of this miR is disregulated in Melanoma (see Table 11)  let-7c, miR-10b, miR-30a-3p, miR30e-3p  collectively underexpressed in a rejection microenvironement(renal acute rejection biopsies ) [70]  but upregulated in our study in adult melanomas | *This is a novel report on miR-30a expression increased in primary melanomas( FFPE) of patients > 60 years stage I-II compared to < 30 years patients.This miR is also differentially expressed between node positive and node negative patients in the two age groups* |
| hsa-miR-29c | Chr1; Location: 1q32.2 | higher | lower |  | COL4A5,COL9A1,COL6A2,COL4A1,COL16A1,ADAMTS7,ADAMTS10,COL6A3,COL5A3,COL3A1,COL11A1,TNFRSF1A,TNIP2 | Potential regulatory function in the stroma surrounding the tumor microenvironment, targets extracellular matrix proteins collagen alpha-chain precursors, disintegrin an metalloproteinase precursors (ADAMS), TNF related proteins |  | *This is a novel report :miR-29c expression is increased in primary melanomas( FFPE) of patients > 60 years compared to < 30 years patients* |
| hsa-miR-99b | Chr9 Location:q34.11 | higher | lower | Calmodulin 2( CALM 2)  mediates the control of  protein kinases and phosphatases; also involved in the  pathway that regulates the centrosome cycle and progression through cytokinesis |  | hsa-miR 99b*, miR-10, miR-125b, miR-30  involved in pathways of local invasion in prostate cancer .These miRs were greater than 2 folds upregulated in prostate cancer with Perineural invasion (PNI), the dominant pathway for local invasion in prostate cancer, than in prostate cancer without PNI [60]. |  | *This is a novel report: miR-99b expression is increased in primary melanomas( FFPE) of patients > 60 years compared to < 30 years patients* |
| hsa-miR146b | Chr5 q34 Location: | higher | lower |  |  |  | Upregulation in the > 60 years group confirms  Upregulation in melanoma with vertical growth pattern and metastatic [32] | Upregulation in melanoma with vertical growth pattern and metastatic [32] |
| **MiR** | **Chromosomal location** | **Expression in adult** | **Expression in young adult/pediatric** | **Known Targets (Entrez,PubMed)** | **Predicted target related to cancer (MiRbase**  **/PicTar** | **Summary of functions** | **Comment melanoma cases in this report and melanoma literature** | **Previously reported in Melanoma vs *this report*** |
| Hsa-miR-211 | Chr: 15; Location: 15q13.3, a locus frequently altered in cancers but not in melanoma | low | higher |  | PLXDC2 ( plexin (Tumor endothelial marker 7-related protein  CCR10 chemokine receptor ( receptor for MCP1,3,4,RANTES,CTAK)  CWF19L1 (CWF19-like 1 Cell cycle control) | Potential Master-immunoregulatory role ? | The expression of hsa-miR-211 is dramatically downregulated in our primary melanoma compared to nevus control and decreases with increasing age | 1.4 fold upregulation of this miR in primary melanoma compared to benign nevi [35]  *miR-211 expression is decreased with increasing age* |
| Hsa-miR-455 | Chr: 9; Location: 9q32 | low | high |  |  | Metabolic processes  differentiation process of brown adypocytes and found to be decreased in muscle tissue where large changes in metabolic capacity occurs |  | *This is a novel report:*  *miR-455 expression is decreased in primary melanomas( FFPE) of patients > 60 years compared to < 30 years patients* |
| Hsa-miR-24 | Chr: 9; Location: 9q22.32 | low | high |  | H2AX | key role in the double-stranded break response, suppresses DNA repair in terminally differentiated blood cells |  | *This is a novel report:*  *miR-24 expression is decreased in primary melanomas( FFPE) of patients > 60 years compared to < 30 years patients* |
| Hsa-miR-944 | Chr 3; Location q28 | low | high |  | c-REL (transactivator member of REL/NFKB family) | Unknown  Potential regulator of NFKB pathway? |  | *This is a novel report:*  *miR-944 expression is decreased in primary melanomas( FFPE) of patients > 60 years compared to < 30 years patients* |
|  |  |  |  |  |  |  |  |  |

NOTE to **Additional file 2**: empty cells in the table signify that incomplete or no information could be found in the literature or reliably reported.
