# Supplementary material for: Microrna profiling analysis of differences between the melanoma of young adults and older adults
Source: J Transl Med. 2010 Mar 19;8:27. doi: 10.1186/1479-5876-8-27 (PMC2855523; doi:10.1186/1479-5876-8-27)
Supplement: Additional file 3 — Supplemental table. Genes deregulated in melanoma and miRs predicted to target these genes [79] [file 1479-5876-8-27-S3.DOC]

**Additional file 3. Genes deregulated in melanoma and miRs predicted to target these genes**

| **Molecule/**  **Chr location/ Mutation** | **Frequency of mutation/deregulation in melanoma** | **Function** | **miR predicted to target the gene coding for this molecule** |
| --- | --- | --- | --- |
| BRAF  Chr:  7; Location: 7q34 | 53-66% (2/3) more frequently in metastatic as compared to primary melanomas  Mutation V600E The mutated BRAF allele was frequently found at an elevated copy number, implicating BRAF as one of the factors driving selection for the frequent copy number increases of chromosome 7q in melanoma [79] | BRAF, a critical serine/threonine kinasey involved in the transduction of mitogenic signals from the cell membrane to the nucleus (MAPK cascade: growth factor receptor > RAS > RAF > MEK > ERK)., is frequently activated by somatic mutation in melanoma | hsa-miR-9? |
| NRAS  Chr: 1; Location: 1p13.2 | 9-29% | The RAS/mitogen-activated protein kinase pathway sends external growth-promoting signals to the nucleus |  |
| RAB-member of RAS oncogene family  Chr:20  Location 20q13.32 | RAB22A has been found to reside in regions of chromosomal breakpoints and have altered/increased expression in melanoma [49] | The protein encoded by this gene is a member of the RAB family of small GTPases. The GTP-bound form of the encoded protein has been shown to interact with early-endosomal antigen 1, and may be involved in the trafficking of and interaction between endosomal compartments | hsa-miR-204 |
| CKIT  Chr: 4; Location: 4q11-q12 | mutation K642E  mutation L576P  increased biol. activity | Expressed by hematopoietic stem cells, mast cells, germ cells, interstitial Cajal cells (intestinal pacemaker cells) and melanocytes.  This single-pass type I membrane protein is the receptor for the ligand stem cell factor (SCF, also known as mast cell growth factor). c-Kit is a receptor tyrosine kinase (RTK): binding of SCF to c-Kit leads to receptor dimerization and activation by autophosphorylation, which is followed by activation of PI3K/Akt and Ras/MAPK pathways | hsa-miR-221  hsa-miR-222 ^32^ hsa-mir-221 could directly inhibit cKit, p27(Kip1) |
| CDKN2A/  P16INKD4/P14ARF  Chr: 9; Location: 9p21  CDK4  Chr: 12; Location: 12q14 | CDKN2A Germline p.G101W inactivating mutation | The CDKN2A locus contains overlapping reading frames that code for p16/INK4A and p14/ARF, two unrelated proteins that have important roles in cell cycle control. p16 inhibits cyclin dependent kinases CDK4 and CDK6 (Rb inhibitors), thus allowing Rb mediated cell cycle arrest. p14 inhibits Mdm2 (p53 inhibitor), thus allowing p53 mediated cell cycle arrest and apoptosis [www.MMMP.org] | ? |
| MET  Chr: 7; Location: 7q31 | Mutation R988C  MET protein (detected by immunohistochemistry) is expressed in 15% of normal nevi and in higher proportion in melanomas (31% to 88%) Activated MET (IHC for phosphorylated MET) is found in melanomas (21%) but not nevi or normal epidermis | This is the receptor tyrosine kinase (RTK) for the hepatocyte growth factor (HGF)/scatter factor (SF). Acts as a disulfide linked heterodimer (extracellular alpha chain + single-pass type I transmembrane protein beta chain; both cleaved from a 170 kDa precursor form). HGF is a multifunctional ligand acting as a mitogen, motogen, and morphogen for many epithelial cells. HGF is physiologically secreted by mesenchymal cells and acts on neighboring epithelial cells through a paracrine loop.  MET overactivity stimulates the invasive growth of cancer cells, inhibits apoptosis and increases their metastatic potential. MET is overexpressed (more frequently) or mutationally activated (less frequently) in a variety of solid tumors. A complex cross-talk exists between MET and other cancer-related receptors (e.g. integrins, FAS, Plexin-B, EGFR/ErbB)(Internet address: www.MMMP.org) | hsa-miR 199a*  hsa-miR 144  hsa-miR 198  hsa-miR 23a  hsa-miR 23b  hsa-miR 34b  hsa-miR 34c |
| SOCS1  Chr: 16; Location: 16p13.13 | SOCS1 plays an important role in the protection against cancer.  In melanoma, SOCS1 hypermethylation increases with advancing clinical tumor stage | SOCS family proteins (CISH, SOCS1-7,) form part of a classical negative feedback system that regulates cytokine signal transduction. SOCS1 is involved in negative regulation of cytokines (IL-6, leukemia inhibitory factor, LIF), Interferon gamma (IFN-g) that signal through the JAK/STAT3 pathway: through binding to JAKs, SOCS1 inhibits their kinase activity using its KIR domain  Defects in the activities of SOCS proteins can contribute to tumor development: | hsa-miR 155, hsa-miR -19a, b  hsa -miR- 331,  hsa -miR-30-a5p  hsa -miR-30b,d,e  Let-7-a,b,c,d,e,i  hsa -miR-98 |
| AKT  Chr: 14; Locations: 14q32.32;  AKT3 E17K mutation | high>25%(Incidence) - activation defined as high expression of pAKT  Primary melanoma: 49%. Metastatic melanoma: 77%  The AKT/PI3K pathway is overactivated in most tumor types (including melanoma), mainly by AKT and/or PI3K overexpression/overactivation and/or by PTEN inactivation. AKT3 is the predominant isoform activated in sporadic melanomas. AKT promotes tumor growth and survival and causes therapy resistance. AKT overexpression converts radial growth to vertical growth melanoma, likely by inducing ROS production and VEGF upregulation. Inhibition of AKT activity has anticancer effects in many preclinical models (including melanoma, which is fostering the development of AKT inhibitors (Internet address:www.MMMP.org) | mTor pathway | ? |
| **PTEN**  chromosome: 10; Location: 10q23.3 | PTEN protein levels are absent/significantly reduced primary melanomas  Oncology: Originally cloned as a tumor suppressor gene (TSG) for brain tumors, PTEN is now known as a TSG for many tumor types, including melanoma(Internet address:www.MMMP.org) | PTEN is a dual-specificity protein and lipid phosphatase. By catalyzing the PIP3 > PIP2 reaction (PI: phosphatidylinositol), antagonizes the activity (PIP2 > PIP3) of PI-3-kinase (PI3K, usually activated by receptor tyrosine kinases upon binding with growth factors) and thus inhibits the activation of AKT (protein kinase B, PKB) a key cell survival factor. In fact PIP3 binding to the PH domain of AKT leads to AKT membrane translocation where AKT is phosphorylated (pAKT) by phosphoinositide dependent kinase 1 (PDK1). | hsa-miR -21  hsa-miR-19  hsa-miR -214 |

**Table 11. Legend**

Molecule/Chromosomal location was obtained from Entrez Gene: [http://www.ncbi.nlm.nih.gov](http://www.ncbi.nlm.nih.gov/)

Mutations, frequency of mutation/deregulation in melanoma and functions were obtained from The Melanoma Molecular Map: <http://www.mmmp.org/MMMP/public/melanomamolecolarprofile> and references reported in the discussion section. MiR predicted to target the gene coding for a particular molecule were obtained from the Pic Tar data base: <http://pictar.mdc-berlin.de/cgi-bin/PicTar_vertebrate.cgi>

MiRs highlighted in yellow were found de-regulated in our study. Gene targeted by miRs differentially expressed in our melanoma specimens are highlighted in blue. “ ?” indicates that the miR is unknown or no miR was found in the literature by the authors.
